# Cross-shore transport and eddies promote large scale response to urban eutrophication

**DOI:** 10.1038/s41598-024-57626-6

**Published:** 2024-03-27

**Authors:** Fayçal Kessouri, Martha A. Sutula, Daniele Bianchi, Minna Ho, Pierre Damien, James C. McWilliams, Christina A. Frieder, Lionel Renault, Hartmut Frenzel, Karen McLaughlin, Curtis Deutsch

**Affiliations:** 1 Department of Biogeochemistry, Southern California Coastal Water Research Project, 3535 Harbor Blvd, Suite 110, Costa Mesa, CA 92626 USA; 2 Department of Atmospheric and Oceanic Sciences, University of California Los Angeles, Los Angeles, CA 90095 USA; 3 Laboratoire d’Études en Géophysique et Océanographie Spatiale, IRD, CNRS, CNES, UPS, Toulouse, 31400 France; 4 School of Oceanography, Seattle, WA 98195 USA; 5 Department of Geosciences, High Meadows Environmental Institute, Princeton University, Princeton, NJ 08544 USA; 6 Present Address: CICOES, University of Washington and NOAA PMEL, Seattle, WA 98105 USA

**Keywords:** Urban eutrophication, Nitrogen coastal transport, Ocean acidification, Deoxygenation, Biogeochemistry, Climate sciences, Ocean sciences

## Abstract

A key control on the magnitude of coastal eutrophication is the degree to which currents quickly transport nitrogen derived from human sources away from the coast to the open ocean before eutrophication develops. In the Southern California Bight (SCB), an upwelling-dominated eastern boundary current ecosystem, anthropogenic nitrogen inputs increase algal productivity and cause subsurface acidification and oxygen (O$_{2}$) loss along the coast. However, the extent of anthropogenic influence on eutrophication beyond the coastal band, and the physical transport mechanisms and biogeochemical processes responsible for these effects are still poorly understood. Here, we use a submesoscale-resolving numerical model to document the detailed biogeochemical mass balance of nitrogen, carbon and oxygen, their physical transport, and effects on offshore habitats. Despite management of terrestrial nutrients that has occurred in the region over the last 20 years, coastal eutrophication continues to persist. The input of anthropogenic nutrients promote an increase in productivity, remineralization and respiration offshore, with recurrent O$_{2}$ loss and pH decline in a region located 30–90 km from the mainland. During 2013 to 2017, the spatially averaged 5-year loss rate across the Bight was 1.3 mmol m$^{-3}$ O$_{2}$, with some locations losing on average up to 14.2 mmol m$^{-3}$ O$_{2}$. The magnitude of loss is greater than model uncertainty assessed from data-model comparisons and from quantification of intrinsic variability. This phenomenon persists for 4 to 6 months of the year over an area of 27,840 km$^{2}$ ($\sim$30% of SCB area). These recurrent features of acidification and oxygen loss are associated with cross-shore transport of nutrients by eddies and plankton biomass and their accumulation and retention within persistent eddies offshore within the SCB.

## Introduction

Rising atmospheric carbon dioxide (CO$_{2}$) concentrations are driving global trends of warming, acidification, and oxygen (O$_{2}$) loss in the ocean, with severe consequences for marine ecosystems. In coastal regions, these global drivers can combine with the effects of eutrophication to exacerbate the decline of pH and O$_{2}$, particularly in shallow and enclosed bodies of water^1–3^. In eastern boundary current ecosystems, wind-driven upwelling drives high biological productivity, routinely exposing shelf waters to low-pH and low-O$_{2}$ conditions^4,5^. The importance of upwelling, along with vigorous coastal circulation, would suggest a minor role for coastal eutrophication in exacerbating these stressors^6^. However, in the Southern California Bight (SCB), an open embayment in the upwelling-dominated southern California Current System,^7,8^ found that anthropogenically enhanced nutrient loads from a coastal population of 23 million people are amplifying primary productivity and subsurface respiration within a 15 km band along the coast, exacerbating acidification and oxygen loss at a rate approaching that of climate change. However, much work remains to better understand the full spatial footprint of the influence of anthropogenic inputs on primary productivity, pH and O$_{2}$ changes in the SCB, and the mechanisms responsible for spatial and temporal variability in these effects.

In open embayments such as the SCB, a key control over the magnitude of eutrophication is the degree to which vigorous currents quickly transport nitrogen away from the coast to the open ocean before eutrophication conditions manifest^6^. Cross-shelf transport and entrapment of nitrogen and newly produced organic matter in persistent eddies represent a physical mechanism, antagonistic to mean currents, that can accumulate organic matter, exacerbating O$_{2}$ and pH declines. Previous work has shown that mean and eddy mesoscale and submesoscale circulation features produce intense biogeochemical variability in the SCB^9^, made more complex by the presence of the Channel Islands^10–12^, abrupt topography, multiple deep basins, and a nonuniform shelf^13^. Offshore, the SCB circulation system is surrounded by the southward branch of the California Current and the northward coastal current, which together generate large standing cyclonic eddies^14^, south and north-west of Santa Catalina Island and north of Nicolas Island. The geography of the shelf favors broad coastal-offshore exchanges, where at some places kinetic energy is more intense (e.g., Palos Verdes peninsula and Point Dume). The island mass effect, enhanced by the wind curl, is the other feature that favors the development of intense eddy circulation in the SCB^11,12^ and amplifies the cross-shelf exchange of energy and material. It also increases the vertical fluxes of ambient nutrients and favors intense seasonal phytoplankton productivity^12^. Nearshore, water residence time varies as a function of basin morphology and shelf and slope width, which causes variability in the relative importance of mean current versus eddy cross-shelf transport.

Anthropogenic inputs of nitrogen and subsequent effects on enhanced nearshore productivity and organic matter accumulation^7^ have the potential to interact with these physical transport processes, resulting in important consequences for the rates of acidification and O$_{2}$ loss in a warming ocean, and the marine organisms that are exposed to them^15^. Point and non-point source discharges to the nearshore of the SCB are partitioned across 75 rivers and 19 ocean outfalls that discharge treated municipal wastewater effluent totaling, on average, 8 million m$^{3}$ d$^{-1}$ of nitrogen-enriched water to the SCB^16^. Nitrogen in river plumes discharges directly to the euphotic zone, while deep ocean outfalls are designed to achieve greater dispersion of contaminants below the euphotic zone^8^. Therefore, physical processes that control water column mixing, and the interaction of these riverine and ocean outfall plumes with mean currents and the eddies and jets that enhance cross-shelf transport, are key to the environmental fate and effects of the anthropogenic nutrients being discharged.

Biogeochemical mass balances analyses are routinely used in coastal numerical modeling to investigate the mechanisms responsible for the spatial and temporal changes in the sources, sinks, and transformations of nutrients, carbon, and O$_{2}$, using principles of mass conservation^17^. Movement between the control volumes used for mass balance reflects physical transport (advection or vertical diffusion), which in essence link the environmental drivers with eutrophication outcomes and its biological consequences. Patterns of change in the mass balance have significant practical importance as they provide coastal managers direct evidence that link the environmental drivers to their effects, especially when those drivers result in effects that occur far away from their point of origin, as is typically the case with eutrophication^18^.

In this study, we assess the fate and consequences of anthropogenic nitrogen loading to the SCB on coastal nitrogen, O$_{2}$, and carbon budgets, utilizing a series of simulations with a regional submesoscale-resolving physical-biogeochemical model (Materials and Methods), updated from previous work^7^ to represent recent conditions (August 2012–November 2017). The model is run at a horizontal resolution of 0.3 km over the SCB, and is forced by realistic atmospheric fields and oceanic boundary conditions. This configuration is part of a suite of “nested” simulations used to propagate basin-scale circulation patterns to the coastal scales where the dispersal and biological utilization of anthropogenic nutrients takes place^8,19^.

Our analysis assesses the change between two simulations. A control simulation (CTRL) does not include any local terrestrial inputs but represents the large-scale effects of the global atmospheric CO$_{2}$ increase (warming and acidification). The anthropogenic simulation (ANTH) includes a synoptic reconstruction of terrestrial inputs of nutrients (nitrogen, phosphorus, silicon, and iron), organic and inorganic carbon (C), and alkalinity from rivers, wastewater outfalls, and atmospheric deposition (Materials and Methods^20^). When the CTRL simulation is subtracted from the ANTH simulation, we attribute the net change to these terrestrial inputs, which represent both natural and anthropogenic sources, of which >95% total N exports originate from point sources of treated municipal wastewater effluent, with a minor fraction from non-point sources^8,20^.

We employ biogeochemical mass balance analyses to investigate the magnitude, spatial extent, and physical processes responsible for changes in patterns of net primary production (NPP), respiration, and remineralization, both inshore and offshore. We then quantify the contribution of these changes to subsurface acidification and O$_{2}$ loss, the basis for subsequent investigations of the effects of eutrophication on loss of suitable habitat for aerobic and calcifying taxa (Frieder et al., submitted).

We previously conducted a model skill assessment, consisting of comparison of model predictions against observations of 1997 through 2001, and demonstrated that the model captures the main distribution and variability of physical and biogeochemical variables in the region with a high degree of fidelity, including nitrogen, O$_{2}$, and carbon system parameters^8^. We update this skill assessment for the 2013 to 2017 period as a check up on performance, then compare how the predicted magnitude of anthropogenic change compared with two quantifiable sources of model uncertainty: (1) differences between observations and model predictions and (2) intrinsic variability that arises from stochastic ocean processes.

## Results

### Effects of land-based nutrient inputs on phytoplankton biomass, oxygen, and pH

**Figure 1:**
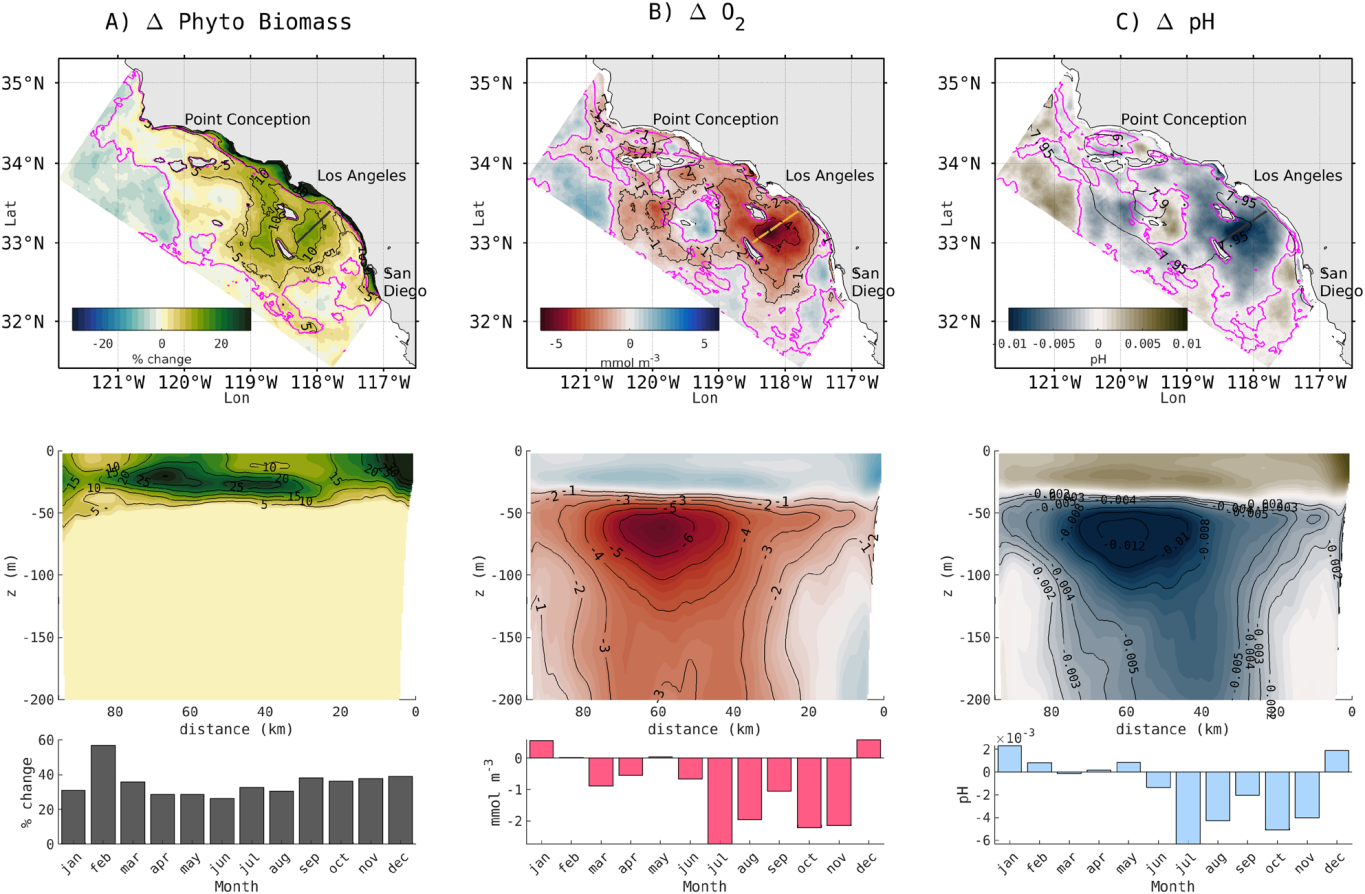
5-year (2013–2017) average difference due to terrestrial inputs. In (**A**) vertically integrated biomass [0-100m], (**B**) subsurface oxygen (at 55m depth), (**C**) subsurface pH (at 55 m depth). Upper panel shows a 2-D view of the 5-year average. Middle row shows the average change across a persistent eddy south of Santa Catalina Island (dark or yellow solid line in the upper panels; distance is measured from NE to SW). Bottom row shows climatological difference in the offshore domain (pink contour in the upper panels) for each variable (intergated biomass [0–100], dissolved oxygen and pH at 55 m depth), shown as a 5-year monthly average.

Bight-wide, the 5-year annually averaged (2013–2017) phytoplankton biomass shows a mean increase of 7 mmol C m$^{-2}$, with values up to 154.4 mmol C m$^{-2}$, relative to the CTRL scenario. Spatially, prominent increases in phytoplankton biomass occur in the coastal band, with more diffuse but ubiquitous effects in the offshore domain, where the average increase reaches 5 mmol C m$^{-2}$ and up to on average 32.8 mmol C m$^{-2}$ over the 5 years (Fig. 1A). This increased biomass occurs throughout the year, with no apparent seasonal cycle (Fig. 1A bottom panel). This increase in biomass shows a strong temporal and spatial coherence with declines in subsurface oxygen and pH (Fig. 1B,C) all over the domain, with spatial averages of 1.3 mmol m$^{-3}$ O$_{2}$, and peak losses up to 14.2 mmol m$^{-3}$ O$_{2}$. Except within 15 km of the coast, average oxygen and pH changes show a seasonality at the subsurface. For pH the average difference is 0.002 and a maximum difference of 0.023 occurring offshore in a 5-year averaged outputs. Offshore, the subsequent pH and O$_{2}$ declines occur most consistently in the summer through late fall.

**Figure 2:**
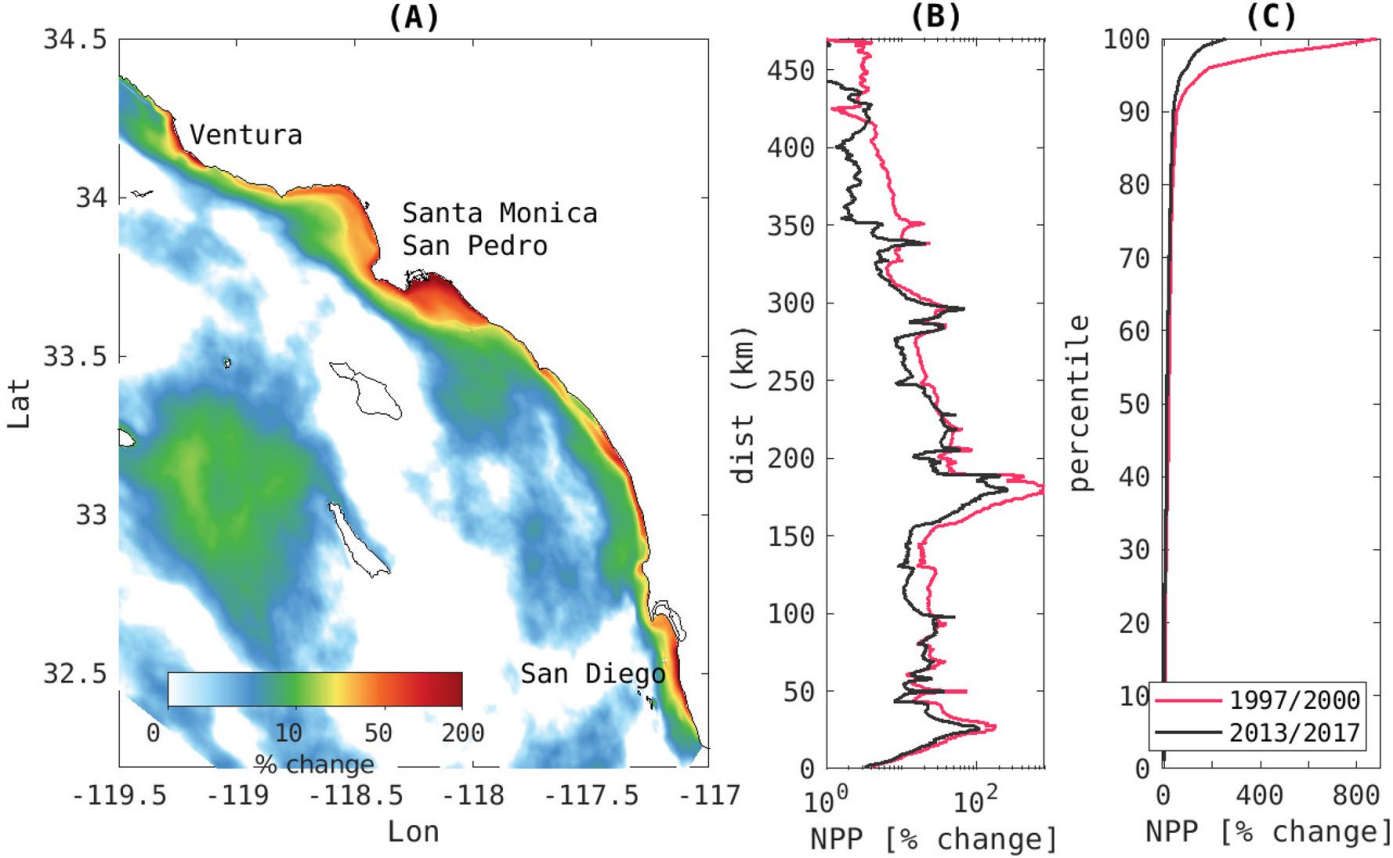
(**A**) is the ANTH-CTRL difference in NPP between the ANTH and CTRL simulations for spring and summer averaged over the 2013–2017 period. (**B**) Alongshore comparison of the averaged difference in NPP in the 15 km coastal band for the 1997–2000 period (red line) and the 2013–2017 period (black line). For each period, the difference between the ANTH and CTRL simulations is shown. (**C**) Comparison of the cumulative probability distribution of the changes shown in (**B**).

Nearshore, within 15 km of the coast, a previous assessment for the period 1997–2000^7^ found that the largest anthropogenic amplifications of net primary production and biomass occurred in the San Pedro and Santa Monica Bays, where productivity increased on average by 80 and 42%, respectively. Relative to the 1997–2000 period, the recent 2013 to 2017 period shows that land-based nutrients have this same persistent effect on elevating NPP and biomass along the coast (Fig. 2A), but with decreased intensity (Fig. 2B). In general, while the mean values and spatial trend have not changed much, the extremes have decreased considerably (Fig. 2C). The largest declines in productivity occurred in the San Pedro Basin, Santa Monica Bay, and nearshore of Santa Barbara. For example, the average temporal peak decreased from 700 to $167\%$ in the San Pedro basin (Fig. 2B). Relative to the earlier simulations (1997–2000), these trends in NPP and biomass generally appear to correlate with reductions to dissolved inorganic nitrogen (DIN) export to the coast due to wastewater nutrient management (Supplemental Fig S1). Peak declines in nutrient loading have occurred in Orange County and on the San Pedro shelf, where the loads from outfalls and rivers have decreased from 83.9 to 63.4 Mg d$^{-1}$ ($-24\%$^20^), while across all regions, coastal DIN export has been reduced by $13\%$^16^.

Offshore (bottom depth > 200 m), the largest effects of land-based nutrients on biomass, oxygen, and pH during 2013-2017 occurred around south Santa Catalina Island and to a lesser extent south of the Channel Islands (Santa Rosa and Santa Cruz Islands) and northeast of San Clemente Island. The area impacted by these biogeochemical changes is approximately 27,840 km$^{2}$ in size. The change in the biomass is focused on the upper 50 m, corresponding to the euphotic zone, with simultaneous increases in O$_{2}$ and pH, signaling the effects of photosynthesis. The maximum change is generally located at the surface in the coastal band, but moves to the subsurface ($\sim$15–40 m) in the offshore domain (e.g., south of Santa Catalina Island, see the cross section in Fig 2A). Below this depth, O$_{2}$ and pH steeply decline, with the largest magnitude change occurring in the depth range of 40–200 m.

### Mass balance

**Figure 3:**
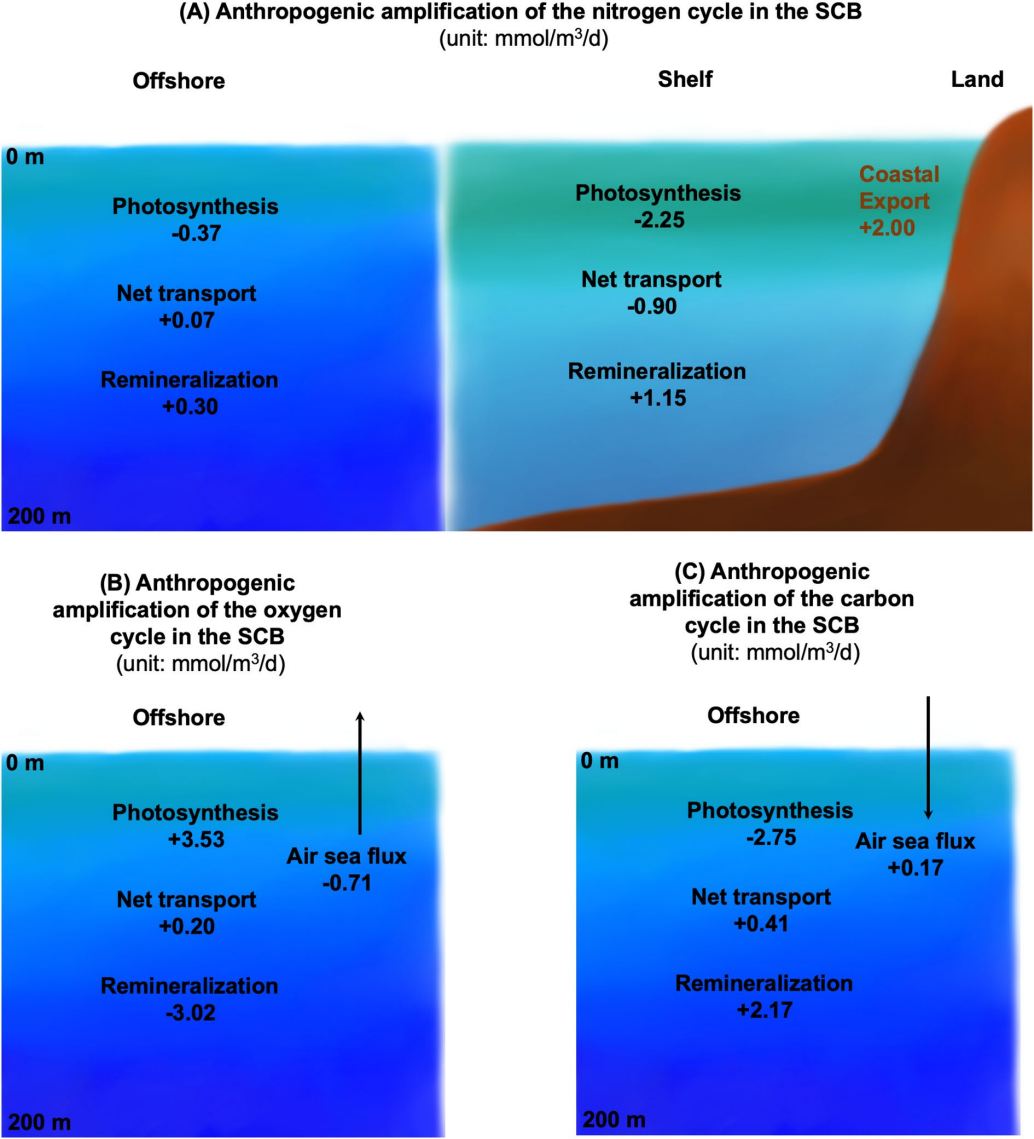
Schematic of the anthropogenic influence (ANTH minus CTRL) of the mass balance averaged 0 to 200 m depth in the (**A**) shelf and offshore domain for DIN (mmol N m$^{-3}$ d$^{-1}$), (**B**) offshore O$_{2}$, and (**C**) DIC. Of note, of the 2.00 mmol N m$^{-3}$ d$^{-1}$ added to the shelf, 2.25 mmol N m$^{-3}$ d$^{-1}$ is taken up by phytoplankton, which is further supported by 1.15 mmol N m$^{-3}$ d$^{-1}$ in remineralized N, while 0.9 mmol N m$^{-3}$ d$^{-1}$ is transported offshore. On average, 2.75 mmol C m$^{-3}$ d$^{-1}$ of plankton carbon is produced offshore, the bulk of which is remineralized/respired, yielding a net O$_{2}$ and DIC loss in the upper 0–200 m.

Eutrophication alters a series of biogeochemical processes that include photosynthesis, respiration, remineralization, and air-sea gas exchange, all of which are mediated by physical transport. These biogeochemical changes can be quantified by a mass balance analysis of DIN, organic carbon (OC), and O$_{2}$. Changes in mass balance of the ANTH relative to the CTRL scenarios are presented here, focusing on the offshore domain (Fig. 3).

Offshore, increased export of OC occurs to deeper waters and to the western model boundary, reflecting the amplification of both new and regenerated primary production in the offshore domain. The intensification of the vertically averaged [0-200 m] photosynthesis ($+$3.53 mmol O$_{2}$ m$^{-3}$ d$^{-1}$, +2.75 mmol C m$^{-3}$ d$^{-1}$), relative to CTRL, is nearly balanced by increased remineralization (+2.17 mmol C m$^{-3}$ d$^{-1}$) and respiration processes ($+$3.02 mmol O$_{2}$ m$^{-3}$ d$^{-1}$). Air-sea fluxes show an amplification of the flux of O$_{2}$ to the atmosphere (loss of -0.71 mmol O$_{2}$ m$^{-3}$ d$^{-1}$) and the uptake of atmospheric CO$_{2}$ (+0.17 mmol C m$^{-3}$ d$^{-1}$), as well as an import of O$_{2}$ and OC by oceanic currents (+0.2 mmol O$_{2}$ m$^{-3}$ d$^{-1}$, $+$0.41 mmol C m$^{-3}$ d$^{-1}$).

**Figure 4:**
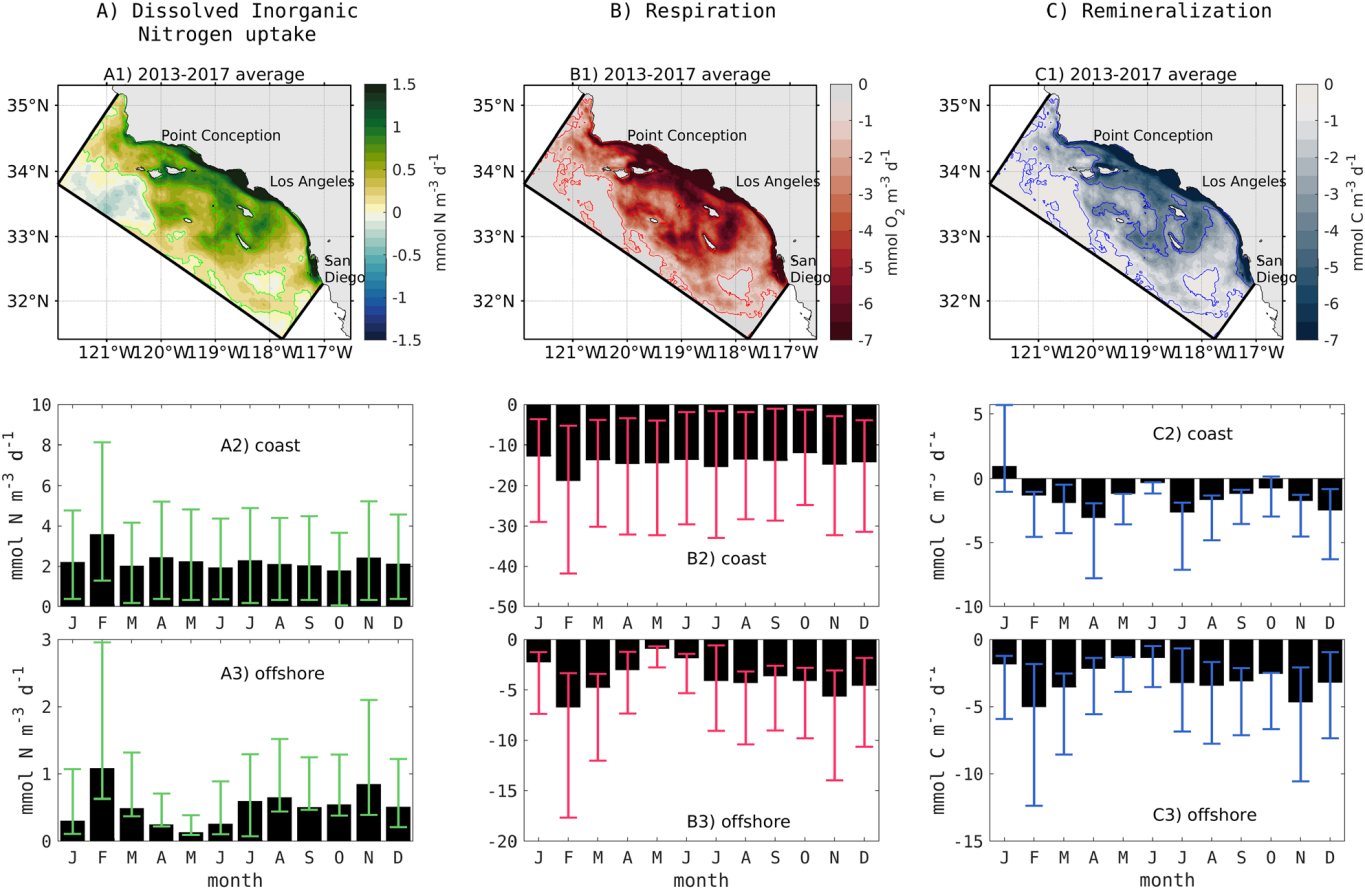
Consequence of eutrophication on biogeochemical processes of (**A**) nitrogen uptake (i.e., NPP) in mmol N m$^{-3}$ d$^{-1}$, (**B**) respiration in mmol O$_{2}$ m$^{-3}$ d$^{-1}$, and (**C**) remineralization in mmol C m$^{-3}$ d$^{-1}$. (A1, B1, C1) Upper panels are 5-year averaged maps, averaged 0-200 m. Light contours on every map highlight 25 and 75 percentiles. Lower panel represents the assessment in a climatological year with 25 and 75 percentiles for each process, (A2, B2, C2) on the shelf (depth < 200 m) and (A3,B3,C3) offshore.

Changes in five-year averaged phytoplankton DIN uptake, respiration, and remineralization, integrated over 0–200 m, illustrate the spatial variability in these rates (Fig. 4A–C), which directly correspond to changes in biomass, O$_{2}$, and pH (Fig. 1A-C). All along the coast on the shelf (depth < 0–200 m), DIN uptake increases by 95%, and respiration and remineralization by 50%.

In the offshore domain, the largest amplification of phytoplankton DIN uptake, respiration, and remineralization occurs southwest and southeast of Santa Catalina Island, parallel to the coast up to the southern coast of the Channel Islands (Santa Rosa and Santa Cruz) and northeast San Clemente Island, as well as northeast San Nicolas Island (Fig. 4A–C). A strong spatial coherence can be seen in the three rates with changes in biomass, O$_{2}$, and pH (Fig. 1A–C). These changes are not necessarily only caused by physical transport of coastal water masses only—local biogeochemical processes can also be a factor. Seasonal climatologies of DIN uptake and respiration on the shelf and offshore reproduce this synoptic variability, but coastal remineralization shows a seasonal variability, which signals a decoupling of coastal production from the locale of its remineralization.

**Figure 5:**
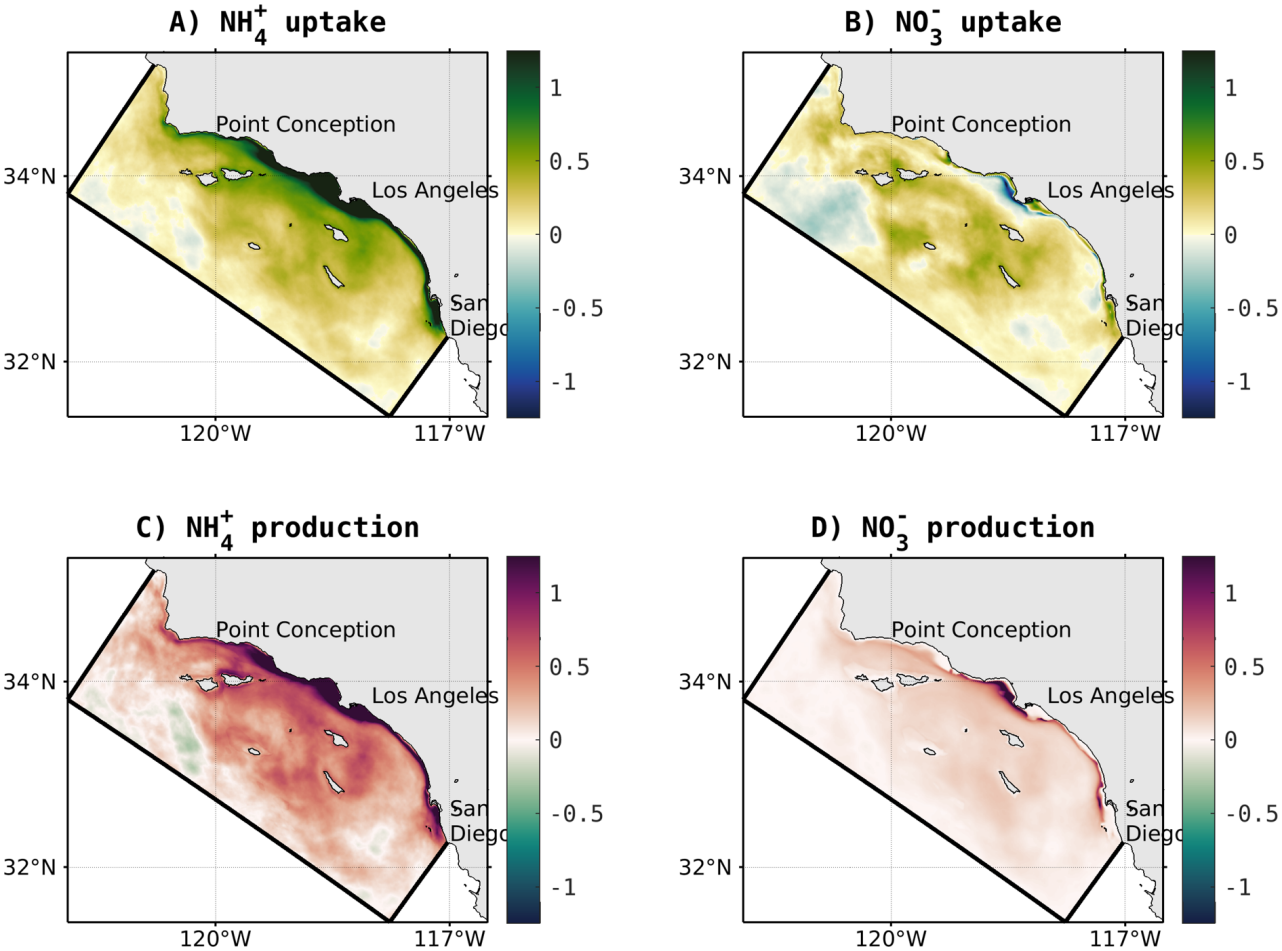
5-year mean of the effects of terrestrial inputs (ANTH minus CTRL) on 0–200 m (**A**) vertically averaged phytoplankton uptake of ammonium, (**B**) the vertically averaged phytoplankton uptake of nitrate, (**C**) ammonium production which includes OC remineralization, biological release, and sediment remineralization, and (**D**) nitrate production via nitrification process. Units are mmol N m$^{-3}$ d$^{-1}$.

This decoupling is also evident in the spatial patterns of NH$_{4}^{+}$ uptake (Fig. 5A), which functions as a tracer for anthropogenic nitrogen inputs, versus recycled NH$_{4}^{+}$ uptake, which is the by-product of remineralization. NH$_{4}^{+}$ uptake shows a broader coastal intensification with increases up to 2 mmol m$^{-3}$ d$^{-1}$, while NH$_{4}^{+}$ recycling shows a greater amplification offshore, with an increase by up to 0.5 mmol m$^{-3}$ d$^{-1}$ (Fig. 5C). NO$_{3}^{-}$ uptake shows variable changes along the coast (Fig. 5B), with reduction in selected nearshore regions, which could reflect inhibition by greatly increased NH$_{4}^{+}$ uptake. Offshore, an overall increase in NO$_{3}^{-}$ uptake is evident. Nitrification shows an enhancement across the domain (Fig. 5D) consistent with the patterns of NH$_{4}^{+}$ regeneration and remineralization.

### Physical processes control fate and transport of anthropogenic nutrients

**Figure 6:**
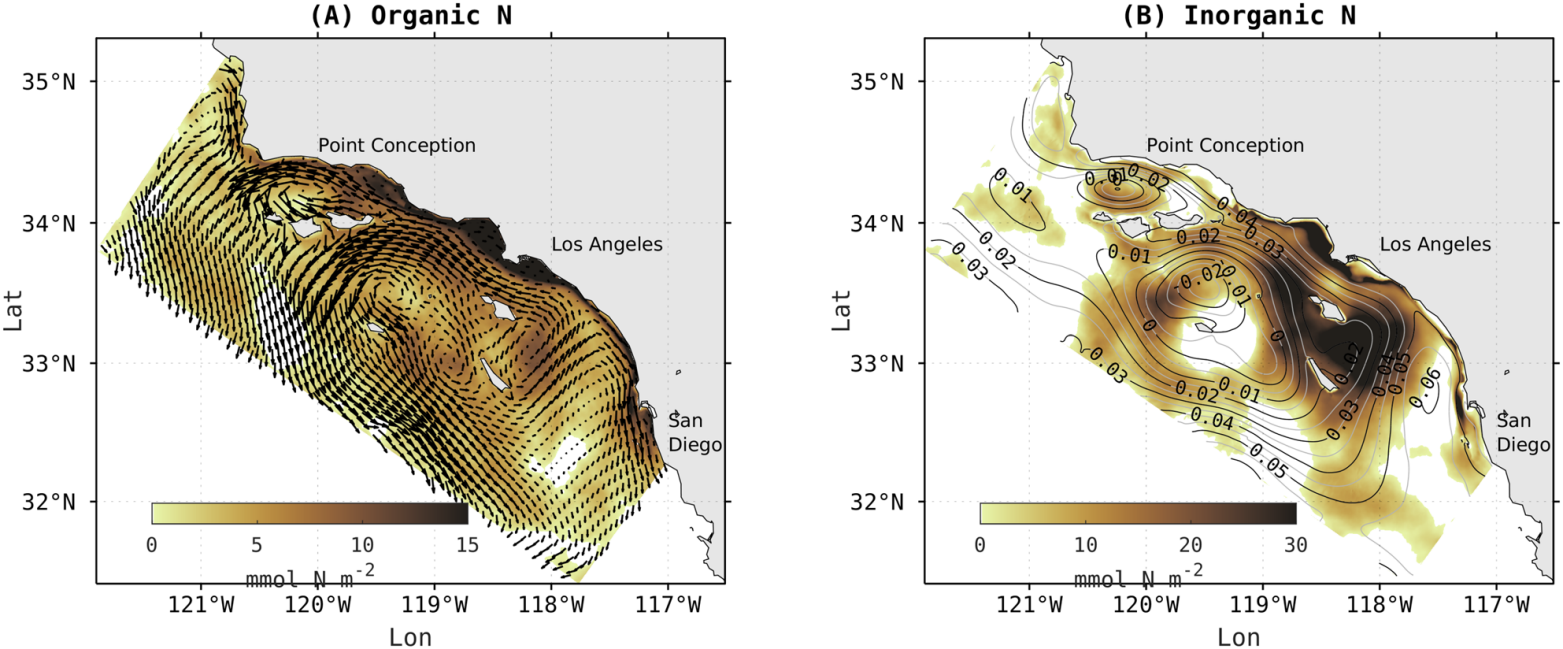
Map of 5-year mean surface currents superimposed on the 5-year mean of the difference between ANTH minus CTRL for (**A**) ON (mmol N m$^{-2}$) and (**B**) surface elevation superimposed on DIN (mmol N m$^{-2}$), integrated over 0–80 m. Surface elevation and mean currents arrows highlight the position of the main currents and the recurrent eddies offshore.

The relative influence of the northern currents and the persistent eddies on the spatial distribution of anthropogenically-enhanced dissolved organic nitrogen (DON) and DIN can be seen by superposing the average currents and sea surface elevation from the ANTH simulation on the net change (ANTH-CTRL) in integrated DON and DIN (Fig. 6A,B). In the offshore domain, anthropogenically enhanced DON is widely spread over the entire SCB. The highest concentration is located inside recurrent eddies southeast of Santa Catalina Island, west of San Clemente, along the southern branch of the northward current onshore, and offshore of the Channel Islands (toward the Islands and toward the southward CCS branch). In contrast to DIN (Fig. 6B), DON (Fig. 6A) does not appear to accumulate inside the eddy of the Channel Islands.

**Figure 7:**
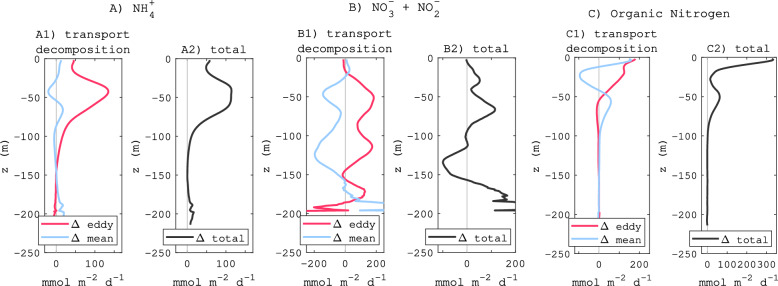
(**A**) is the change (ANTH-CTRL) of ammonium cross-shore physical flux from shelf (depth < 0–200 m) to offshore (positive sign). (**B**) is for nitrate and nitrite. (**C**) is for ON comprising DON, phytoplankton, and zooplankton biomasses. The flux is temporally averaged over 5 years and spatially averaged along the 200 m isobath from San Diego to Santa Barbara. Units are mmol N m$^{-2}$ d$^{-1}$. All fluxes are decomposed into mean and eddy components using a 1-month temporal window.

The 5-year averaged cross-shore physical flux of nitrogen forms (NH$_{4}^{+}$, NO$_{3}^{-}$ plus NO$_{2}^{-}$, and organic nitrogen) from the shelf to offshore (quantified as the transport across a vertical surface that follows the 200 m isobath) reveals multiple pathways of export for each component with a distinct vertical structure (Fig. 7A–C). For instance, NH$_{4}^{+}$ is exported mostly by transient eddies in the upper 80 m, while the cross-shore flux by mean currents is negligible (Fig. 7A). NO$_{3}^{-}$ fluxes show a balance between export by eddies and import by the mean current (likely reflecting upwelling; Fig. 7B). DON offshore fluxes show an important influence from both the mean current and transient eddies, both of which intensify offshore at the surface (Fig 7C).

**Figure 8:**
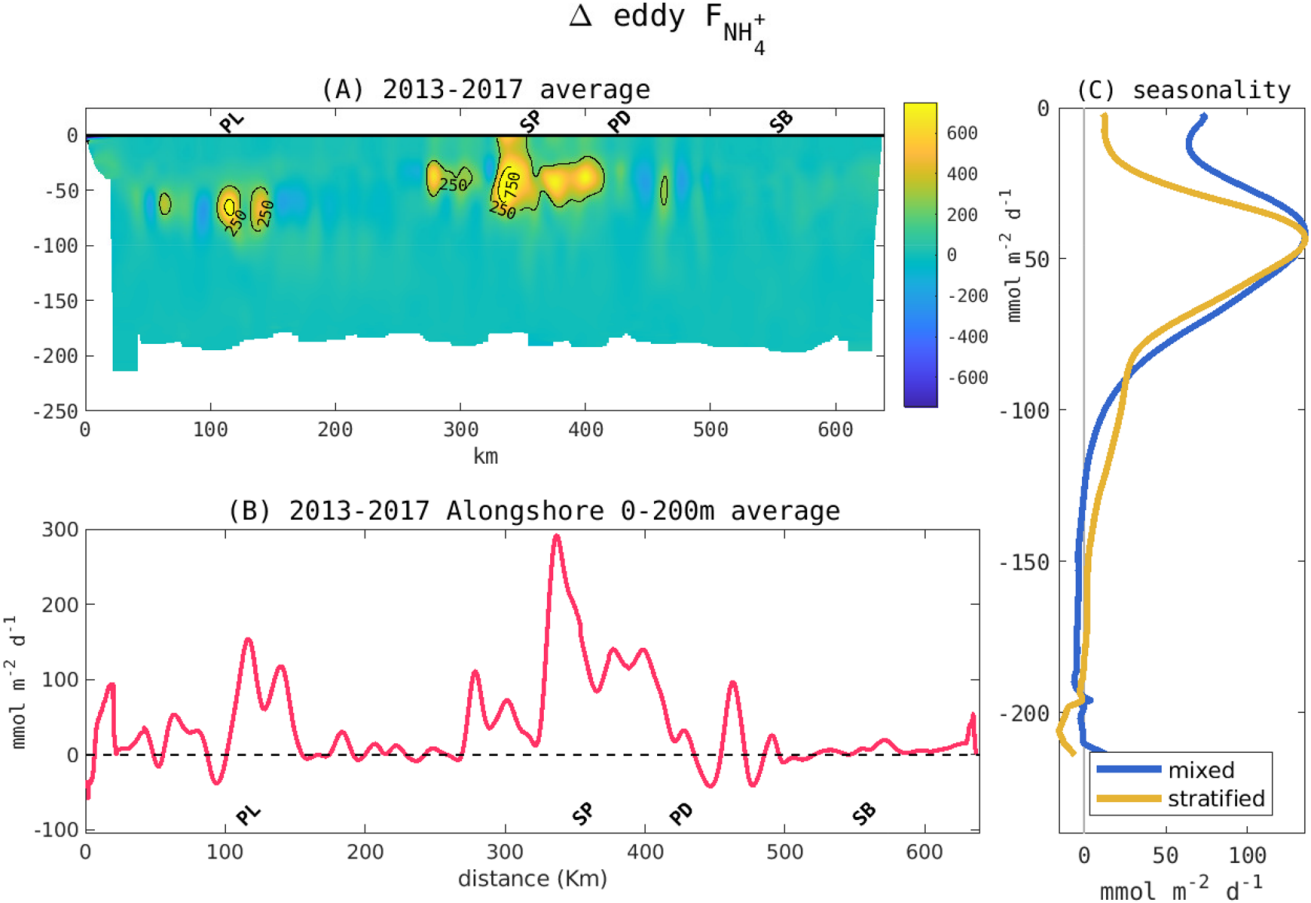
(**A**) Alongshore section showing the cross-shore physical total flux of ammonium at 200 m depth limit. (**B**) is the vertically integrated eddy flux and (**C**) is the seasonality of the eddy flux. Mixed season averages all the months between October and March. Stratified season averages all the months between April and September.

Alongshore, NH$_{4}^{+}$ is exported across the shelf at rates that are proportional to the magnitude of coastal export by outfalls, which are especially notable on the San Diego, Palos Verdes, and Santa Monica shelves (Fig. 8A,B). This alongshore distribution of the flux is also in agreement with an evaluation made using Lagrangian parcels (detailed analysis and results are available in SI). NH$_{4}^{+}$ is exported mainly in deeper layers (30–80 m) during April through June when the water column is stratified, but it can be exported offshore at the surface during periods of weaker stratification (July to March; Fig. 8C). The cross-shelf flux of NH$_{4}^{+}$ by transient eddies varies regionally along the coast and is enhanced by circulation characteristics with intense strain associated with anticyclonic (e.g., along Santa Monica Bay) or cyclonic eddies (e.g., south San Diego coast). A detailed analysis of the transient eddies’ characterization is presented in SI.

### Model uncertainty assessment

**Figure 9:**
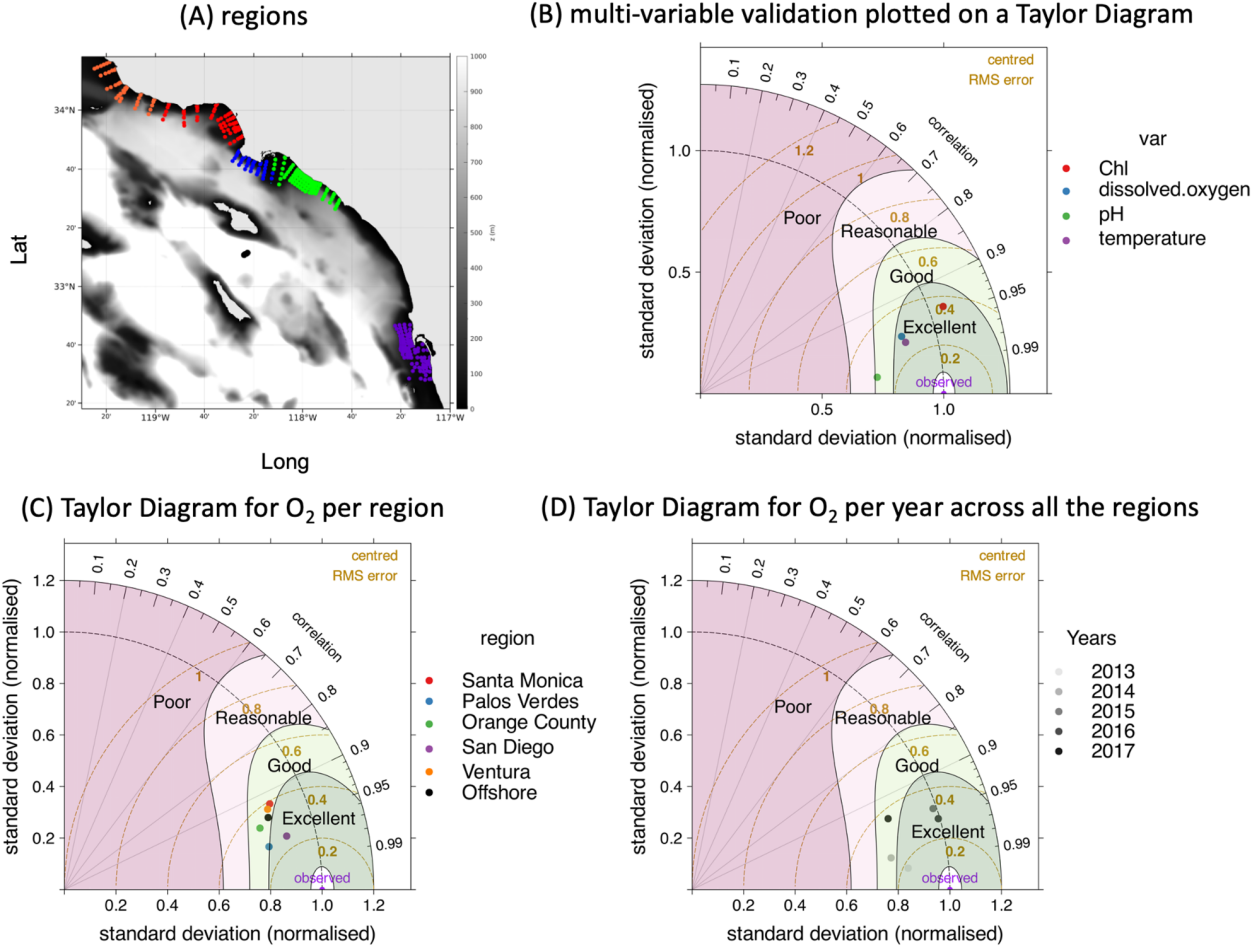
(**A**) Location of the observational stations used in this validation. The gray background represents the bottom topography. (**B**) Taylor diagram illustrating a validation for chlorophyll-a, dissolved oxygen, pH and temperature across all the regions. (**C**) same as B for oxygen, now plotted by sub-region. (**D**) same as B for offshore dissolved oxygen plotted by year across all the regions.

Given that this is a model based assessment, the question is whether the modeled signal of anthropogenic change emerges from the noise of quantifiable sources of model uncertainty. The performance check demonstrates that the SCB ROMS-BEC model continues to exhibit excellent to good performance in capturing key gradients (seasonal, vertical, spatial) of both coastal and offshore temperature, Chl-a, pH and O$_{2}$ during the time period of this evaluation (2013–2017; Fig. 9A). Offshore model performance, particularly in the regions identified for recurrent O$_{2}$ and pH loss, south of Catalina Island, is either equal to or better than in the coastal regions (Fig. 9B). The model demonstrates high performance over the years for the regions identified for recurrent O$_{2}$ and pH loss (Fig. 9C).

**Figure 10:**
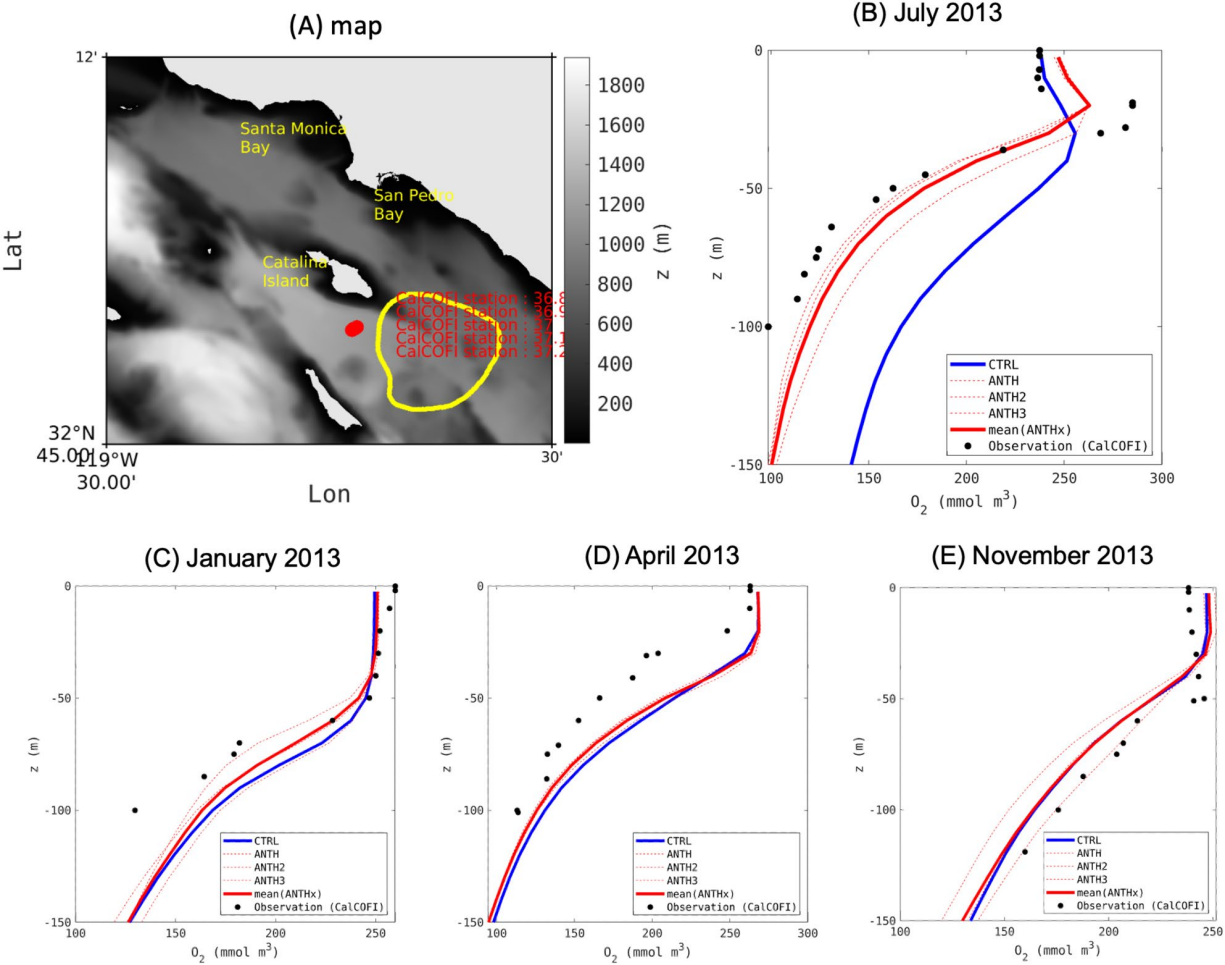
(**A**) A map showing the location of the offshore stations and the yellow contour south of Catalina Island used to plot the time series in Fig. 11. The gray background represents the bottom topography. (**B**) Vertical profile of dissolved oxygen averaged in July 2013 for the CTRL scenario, the averaged ANTH1, ANTH2, ANTH3 scenarios in solid red line, including the additional uncertainty runs as dashed red lines, and the observations taken from the CalCOFI station shown by red dots in panel (A). (**C**–**E**) are the same as (**B**) for January, April and November when ANTH and CTRL do not emerge from the uncertainty runs. The goal of this seasonal validation is to show the realism of the oxycline and dissolved oxygen concentrations throughout all other CalCOFI monitoring periods in 2013.

At the offshore locations of recurrent O$_{2}$ (and pH) loss, the magnitude of changes due to input of anthropogenic nutrients (ANTH-CTRL) in the July–December period was substantially larger than data-model differences and intrinsic variability (Fig. 10B). For example, in July 2013, ANTH-CTRL (-65.49 mmol m$^{-3}$ O$_{2}$) at 50-m was 8-fold greater than data-model difference (8.84 mmol m$^{-3}$ O$_{2}$) and 3-fold greater than the range of intrinsic variability (17.84 mmol m$^{-3}$ O$_{2}$) at this CalCOFI station. While the data-model differences are noticeably variable vertically and seasonally (Fig. 10B–E), the 5-year average data-model difference from 50 m to 150 m was 6.4 $+/-$ 17.2 mmol m$^{-3}$ O$_{2}$ for the periods of typical seasonal hypoxia (July–December). From this exercise, we can conclude that the model “signal” of anthropogenic change emerges from quantifiable noise, lending greater scientific confidence in the results. Of course, these results are scale dependent, but even after averaging across a larger area (Fig. 10A), we see strong evidence that model intrinsic variability is not greater than the signal of anthropogenic change (Fig. 10B).

Because the assessment of intrinsic variability can be at any temporal or spatial scale, we extend its use to further contextualize the analysis of mass balance. For example, in 2013, the average daily change caused by anthropogenic sources in respiration varies from 17 to 21 to 23 mmol m$^{-3}$ s$^{-1}$ O$_{2}$ in the three scenarios on the layer that span between 0-200 m depth. This presents a maximum uncertainty of 6 mmol m$^{-3}$ s$^{-1}$ O$_{2}$, which represents a 5% uncertainty. The anthropogenic signal with a minimum of 17 mmol m$^{-3}$ s$^{-1}$ O$_{2}$) emerges from the uncertainty range. Regarding the uptake of nutrients by coastal phytoplankton, in 2013, the uncertainty of intrinsic variability represents about 5% of the annual signal (about 0.08 mmol m$^{-3}$ s$^{-1}$ N). The maximum uncertainty across the three scenarios does not exceed 0.36 mmol m$^{-3}$ s$^{-1}$ N relative to 1.91 mmol m$^{-3}$ s$^{-1}$ N change caused by anthropogenic sources. This is another confirmation that the anthropogenic signal emerges from the range of uncertainty by intrinsic variability in coastal biogeochemical processes.

## Discussion

A key control on eutrophication in coastal ecosystems is the degree to which vigorous coastal currents transport nitrogen quickly from the coast to the open ocean before eutrophication develops. Previous work^7^ demonstrated that, in the SCB, an upwelling-dominated eastern boundary current ecosystem, coastal anthropogenic nutrient loading results in increased algal productivity and subsurface acidification and oxygen loss within a nearshore band (<15 km). With an expanded analysis of more recent simulations, we found that subsurface acidification and O$_{2}$ loss, resulting from this enhanced productivity and respiration, also significantly affect the offshore regions of the SCB. The magnitude of these changes can emerge from quantifiable estimates of data-model uncertainty and intrinsic variability. We document recurrent offshore hot spots of subsurface acidification and O$_{2}$ loss located 30–90 km from the mainland, including the deep basin south and west of Santa Catalina Island, south of the Channel Islands, and north of San Nicolas Island. In total, these hot spots occupy an area of 270,80 km$^{2}$ on average over the five-year simulation period, with vertical pH decline and O$_{2}$ losses of up to 6% persisting 4–6 months of the year. These recurrent features offshore appear to be associated with persistent cyclonic eddies that trap and retain nutrients and organic material originating from the coast, which in turn intensifies local primary production and remineralization.

Transport of DIN and DON from the coastal to the offshore region occurs by a combination of mean currents and transient eddies. The existence and role of these eddies in the transport of upwelled material have been documented for CCS^21,22^. The latter consist of intensely variable currents, jets, and filaments that last a few days and are generated by a combination of baroclinic and barotropic instabilities, and interaction between tides, mean currents, and topography^23^. These transient features detach from the main coastal northward current to export nutrient-enriched coastal waters offshore, where they can accumulate in retentive features. Mass balance and physical transport analyses demonstrate that cross-shelf transport represents an important mechanism of nutrient delivery, antagonistic to the dilutive and dispersive effect of the main currents, that can favor accumulation of organic carbon and nutrients in retentive circulation features, exacerbating acidification and O$_{2}$ loss offshore. This novel finding highlights the importance of coastal circulation features in enhancing the vulnerability of eastern boundary current ecosystems to human coastal nitrogen exports^16^. Given that 48% of the global wastewater discharge receives no treatment^24^, this suggests a pressing problem that requires further investigation in global assessments of coastal acidification and O$_{2}$ loss^25^.

Physical processes such as persistent currents and transient eddies are the principal drivers for these phenomena, acting synergistically or antagonistically to modulate the spatial and temporal patterns of anthropogenic nutrient dispersal, primary production, and respiration changes. We find that organic matter is exported cross-shelf at the surface by the main currents when the ocean is more stratified (spring and summer), but this export can occur deeper by eddies during well mixed periods, which typically occur in fall and winter. In contrast, NH$_{4}^{+}$, the best tracer of anthropogenic nitrogen sources in the region^16^, is exported mostly by transient eddies in the upper 80 m, at rates proportional to local rates of land-based nitrogen discharges, while the cross-shore flux by mean currents remains small.

The rates of decline documented in this study and that of Kessouri et al.^7^ approach global open-ocean rates of oxygen loss and acidification since the preindustrial period (i.e. 5 mmol O$_{2}$ m$^{-3}$ and 0.1 pH units^26,27^). These local and global changes are superimposed, and both are generally enhanced in coastal waters^28–31^; in combination, they can lead coastal ecosystems more rapidly toward ecological tipping points. Because of temporal and vertical habitat compression in summer to late fall, which is most severe in the 50-150 m depth range, epipelagic−dwelling life forms are the most likely to be impacted, including calcifying zooplankton (e.g., pteropods), larval life stages of echinoderms, crustaceans, and mollusks, plus a variety of fish and invertebrates that live at the limit of their aerobic habitat^32–35^. Even relatively small reductions in O$_{2}$ and pH can drive habitat compression, a topic that is further investigated in a companion study (Frieder et al., submitted).

Our analyses show a consistent, year-round amplification of NPP by approximately 25% in average across the domain and up to 200% in the Los Angeles area during the 2013-2017 period, coupled with seasonal reductions of subsurface oxygen and pH up to 15 mmol m$^{-3}$ and 0.02, respectively. These values are lower than, but still comparable to changes in hindcasts of SCB waters for the 1997–2000 period^7^. Over the past two decades, total nitrogen exports have declined $8\%$, with subregional declines up to $83\%$, focused on the Los Angeles and Orange County shelf, where most of the nitrogen export to the SCB occurs. NPP shows a clear decline in this subregion, but interannual variability of ocean state^36^ is clearly a strong modulator of the response of the system to N reductions^7^, which in this analysis cannot be disentangled. Idealized scenarios testing efficacy of nitrogen reduction found that during high productivity years (e.g., 1999, 2017), the efficacy of anthropogenic nitrogen reductions in changing NPP was dampened relative to low productivity years by approximately an order of magnitude^37^, driven by anomalous oceanic−atmospheric interactions that lead to shallower nitracline and nutrient enrichment of the surface mixed layer^38^. Thus, while coastal ecosystem eutrophication responses to point sources are more efficient than to non-point sources^39^, considerable complexities exist in the responses of coastal ecosystems to eutrophication^18,40^, leading to uncertainty in the outcome of local nutrient management strategies.

Shelf (depth < 200 m) NPP increases were roughly five times those of offshore areas, but the magnitude of O$_{2}$ and pH loss only doubled along the shelf, leading to questions about the mechanism for this disproportionate response. Three possible processes were investigated through mass balance: (1) coastal anthropogenic nitrogen is exported cross-shelf to increase new production offshore, (2) OC (and associated nitrogen) produced inshore is exported offshore, and (3) OC accumulates and remineralizes in offshore eddies. We found evidence that all three processes are occurring and altering subsurface OC and O$_{2}$ cycles, but cross-shelf export of OC and entrapment by persistent eddies was largely responsible for the disproportionate response. Persistent cyclonic eddies can be disproportionately more productive than adjacent waters fueled continuously by frontal enrichment by deeper nutrients. They have previously been recognized in other regions as cores for marine production and carbon export, such as in the Sargasso Sea^41^, the Alboran Sea and Algerian current^42,43^. More importantly, the SCB persistent eddies can be isolated for a few months during which anthropogenically intensified productivity can play an important fertilizing role, amplifying carbon export and respiration in subsurface layers, and leading to cumulative O$_{2}$ and pH declines. While coastal waters are generally more productive, they are shallower and more ventilated. Thus, the relationship between surface productivity and subsurface dissolved inorganic carbon (DIC) increase and O$_{2}$ loss may be more substantially altered by transport, air sea, and sediment fluxes, and respond in a non-linear way to nutrient inputs. The Santa Barbara Channel eddy differs from this pattern: DIN appears to accumulate, but OC does not. Therefore, anthropogenic effects on subsurface pH and O$_{2}$ appear to be reduced in this region. This unique system is saturated by a continuous supply of nutrients supported by submesoscale eddies arising from a vigorous island mass effect, i.e., the interaction of winds, currents, and topography^12^.

Our findings augment the evidence that suggests coastal eutrophication in upwelling-dominated ecosystems can have a significant effect on acidification and oxygen loss. Furthermore, they point to two important roles of ocean circulation in driving eutrophication not only along the coastal band but also in offshore waters. First, the coastal circulation effectively transports DIN and OC offshore along preferential pathways associated with vigorous currents and transient eddies. Second, offshore retentive features such as persistent cyclonic eddies appear to trap anthropogenic nutrients, amplifying local primary production, biomass accumulation, export, and remineralization. This amplification, in turn, leads to cumulative effects of the subsurface pH decline and O$_{2}$ loss localized within these features. These co-occurring stressors, in combination with marine heatwaves, are likely to result in habitat compression for a variety of marine organisms, a topic further explored in Frieder et al. (submitted).

Uncertainty exists in these predictions and we endeavoured to quantify that uncertainty to improve confidence in model applications. The previous model performance assessment for both physics and biogeochemistry was good to excellent^8,19,44^. Using the same methodology, our updated model performance during the “check up” of the 2013-2017 period was as good or better than previously documented performance, lending credibility to its utility in scientific investigations of urban eutrophication in the SCB. Furthermore, when placing the findings of our assessment of anthropogenic change and intrinsic variability on the same space-time scales as the performance assessment, we see that the modeled signal of anthropogenic change far exceeds the noise of model uncertainty from either data-model differences or from modeling the “within-domain” chaotic nature of eddies. Although the model shows a consistent signal for the eddy transport of anthropogenic nutrients and organic matter, extending intrinsic variability simulations for multi-annual periods and including both CTRL versus ANTH would be useful to refine our estimates^45^ and ultimately would be useful as a means to limit interpretation of any change assessment. Observational programs tend to under-sample offshore regions. Thus, future work could focus on expanding observational programs beyond nearshore waters, to provide a stronger basis for model validation in the locations where the greatest rates of subsurface pH and O$_{2}$ loss are projected to occur.

Other sources of uncertainty exist in this assessment. For example, we know that the interannual variability of the ocean state is a strong control over the effects of anthropogenic nitrogen loading on the NPP. Years with naturally higher oceanic productivity with a shallow nitracline and mixed layer experience a relatively lower anthropogenic response than a nonproductive year (e.g., 1998 El Niño), when the water is warmer and more stratified^7^. During the period 2013 to 2017 examined in this study, we capture years that are climatically similar, with the exception of the transition year 2013. The period is marked by a marine heat wave from 2014 to 2016, which limited mixing between deep and surface waters. Despite this interannual variability in ocean state, our analysis demonstrates consistency in the amplification of the main mass balance terms to the anthropogenic loading over multiple years. Seasonally, the anthropogenic response at the NPP is stronger in summer due to the vertical structure of the water column and the timing of winter mixing and phytoplankton blooms. The transport of newly formed organic matter by cross-shore eddies occurs after the bloom season, and subsurface anthropogenic nutrients that have been mixed into the surface in winter and spring are also transported when stratification is stronger.

The two scenarios upon which this work was conducted represent an all-or-none approach; however, terrestrial inputs reflect a complex mixture of point, non-point, and natural sources, responding in a nonlinear fashion to variable coastal circulation. Some outfalls and rivers may play a disproportionate role in coastal eutrophication, since the location of discharge (e.g., near headlands that cause wakes^10^) affects mixing and dispersion by persistent currents and eddies. Further research is needed to understand the relative contribution of specific outfalls and rivers along the coastline, including the import of Mexican transboundary sewage flows^46^, the effects of which are under-represented in this work.

Finally, work is needed to unravel the relative contribution of natural variability and climate change, which includes downscaling and properly resolving fine-scale pH and O$_{2}$ variability under future climate scenarios^35^, ranging from high emissions to strong mitigation, to project the range of possible pH, O$_{2}$ and warming stressors that coastal ecosystems will experience^47^. This would provide a refined understanding of the relative importance of local anthropogenic loading to acidification and O$_{2}$ loss, and how much time management actions could confer increased coastal resilience, given the accelerating acidification and oxygen loss from climate change^29,47^. This type of knowledge represents a key context for further information on the role of coastal water quality management in the adaptation to climate change.

## Materials and methods

We utilized the Regional Ocean Modeling System (ROMS^48,49^), coupled to the Biogeochemical Elemental Cycling model (BEC^50^), parameterized for the California Current System (CCS^19,44^) to simulate the effect of land-based inputs of nutrients, carbon, and iron on coastal ocean biogeochemistry and the lower trophic ecosystem over a 20-year period. We evaluated spatial and temporal changes in the mass balance of nitrogen, carbon, and oxygen, and investigated the specific processes responsible for the nearshore versus offshore fate and effects.

### Oceanic model and configuration

A detailed description of the ROMS-BEC model setup and configuration is presented in Kessouri et al.^8^, but summarized here. BEC is a multi-element (C, N, P, O, iron (Fe), and silica (Si)) and multi-plankton model that includes three explicit phytoplankton functional groups (picoplankton, silicifying diatoms, and N-fixing diazotrophs), one zooplankton group, and dissolved and sinking organic detritus. Remineralization of sinking organic material follows the multi-phase mineral ballast parameterization of Armstrong et al. (2001), and sedimentary processes have also been expanded. Particulate organic matter reaching the sediment is accumulated and remineralized with a time scale of 330 days, to provide a buffer between particle deposition and nutrient release. The ecosystem is linked to a carbon system module that tracks dissolved inorganic carbon and alkalinity, and an air-sea gas exchange module based on the formulation of Wanninkhof, 1992^51^.

The SCB model domain, which extends from Tijuana/Mexico to Pismo Beach (U.S. Central California coast) and about 200 km offshore, is part of a one-way nested configuration. Model nests scale from a 4 km horizontal resolution configuration spanning the entire CCS^19,44^, to a 1 km resolution grid covering much of the California coast^52^ (latitude < 40.25$^{\circ }$N), to a 0.3 km grid in the SCB^8^, where our investigations of local anthropogenic inputs were focused^7^. This grid, shown in Fig. 1A, is composed of 1400 $\times$ 600 grid points, with 60 $\sigma$-coordinate vertical levels using the stretching function (with the following parameters: $\theta _{s}$ = 6, $\theta _{b}$ = 3, and $h_c$ = 250 m) described in^49^. The model is run with a time step of 30 s, and outputs are saved as 1-day averages and the physical and biogeochemical diagnostics and fluxes are saved as 1-month averages.

**Figure 11:**
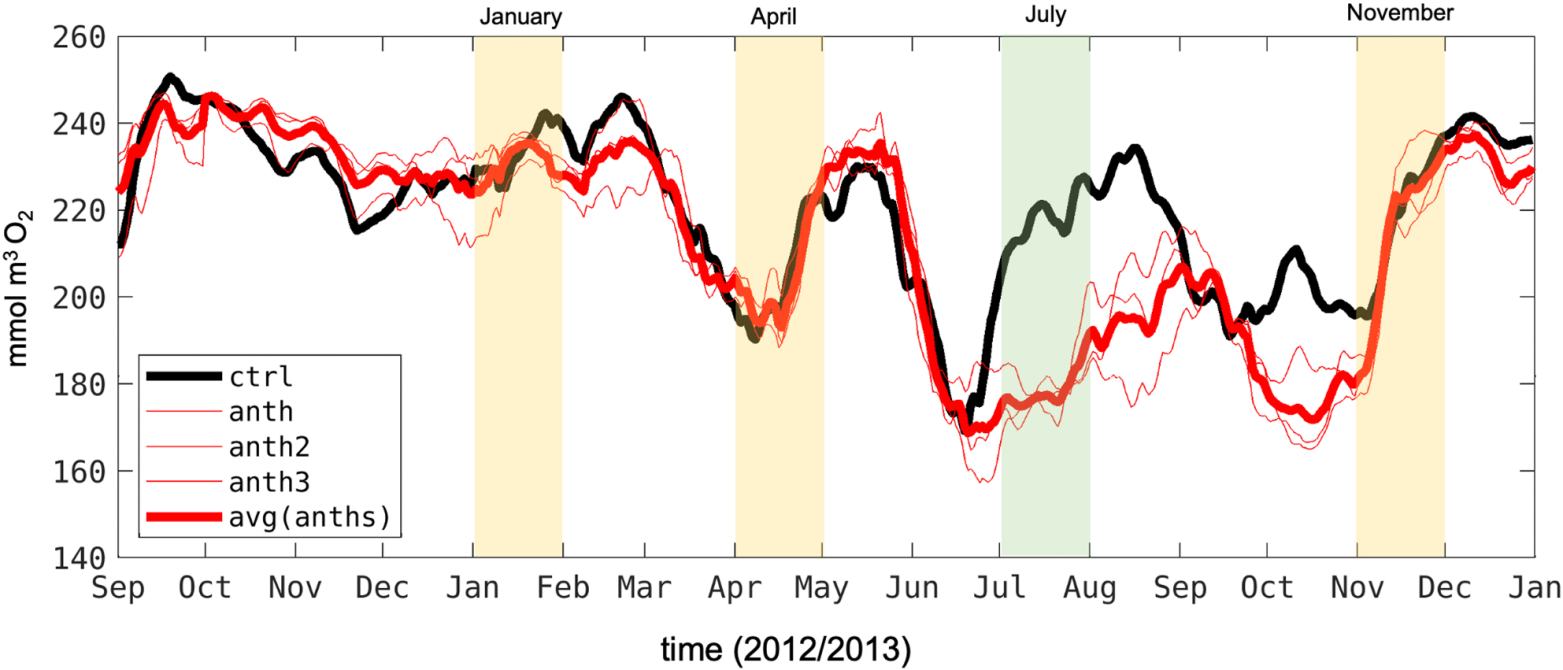
Time series of the concentration of dissolved oxygen in the subsurface averaged in the yellow area south of Catalina Island. The yellow area represents a repeating portion of the Bight where oxygen and pH were depressed by anthropogenic sources during the study period. The colored shared are times where the model was averaged for comparison with CalCOFI data shown in Fig. 10. The yellow shades demonstrate the time period when ANTH and CTRL do not emerge from each other. The green shade is a period where the ANTH signal emerges from the uncertainty.

The 1-km model is run for the period 01/1997–12/2017, after a spin-up of 4 years. Hourly surface momentum, heat, and freshwater fluxes are derived from a 6-km resolution Weather Research and Forecast^53^ atmospheric simulation^44^ using bulk formulae modified to include a wind-current coupling parameterization necessary to attain more realistic oceanic submesoscale circulation^54,55^. ROMS-BEC has been validated for atmospheric forcing, physics, and biogeochemistry, including O$_{2}$, carbonate saturation state, primary productivity, and hydrographic parameters at a West Coast-wide scale for the period of 1995–2010^19^ and, within the SCB where anthropogenic nutrients inputs influence coastal eutrophication, at both nearshore and offshore spatial scales for the years of 1997–2001^8^. More information on the model setup, forcing and coast wide validation is provided in other works^8,19,44^. Analysis and Figs. 1, 2, 4, 5, 6, 7, 8, 9, 10, 11 and S4 have been generated using the version 2022 of the programming software Matlab^56^ and all the maps have been created using the mapping toolbox created by Piretzidis et al.^57^.

### Model scenarios

A set of two simulations are used to quantify the effect of land-based nutrient export on nitrogen, oxygen, and carbon cycling. A first control simulation (CTRL) does not include any terrestrial inputs and thus represents only natural ocean cycles of nutrient, carbon, and oxygen, with the effects of atmospheric deposition and global CO$_{2}$ superimposed. The second includes both natural oceanic cycles of nutrients that represent these same base conditions as the CTRL scenario, to which inputs from terrestrial sources of nutrients, C and Fe are added (ANTH). These terrestrial sources, 98% of which are anthropogenic and 95% of which are point source in origin^16^, consist of ocean discharges of treated wastewater discharged to 19 ocean outfalls from publicly owned treatment works (POTW), and point, non-point, and natural sources in riverine surface water inputs from 75 coastal confluences.

Previous work^7^ quantified the effects of land-based nutrient inputs during 2/1997–01/2001. In this work, we extend the model to recent periods (08/2012–11/2017). Over this period, Bight-wide wastewater volumes have declined by 33% and coastal nitrogen loads by 14%^16^. The coast of Los Angeles and Orange Counties including Santa Monica Bay is the location with the highest volume flux of nitrogen (6.78 mmol m$^{-3}$d$^{-1}$ of total DIN, 85% of which is in the form of NH$_{4}^{+}$), representing 52% of the total SCB discharges. Farther south, the San Diego coast currently receives coastal DIN exports of 4.24 mmol m$^{-3}$d$^{-1}$, 74% of which was NH$_{4}^{+}$. Other subregions such as Ventura/Santa Barbara and south Orange County/Northern San Diego receive appreciably less DIN, with higher proportions of NO$_{3}^{-}$ relative to NH$_{4}^{+}$ (<1 mmol m$^{-3}$d$^{-1}$).

### Biogeochemical mass balance methods

Biogeochemical mass balance analyses are routinely used to investigate the mechanisms responsible for the spatial and temporal changes in the net sources, sinks, and transformations of carbon, nitrogen, and oxygen using principles of mass conservation. Conceptually, mass balances are conducted on units of fixed volume (i.e., a “box”), from which constituents can enter (sources) or leave (losses). Because all the grid cells are adjacent in the model, sources to one box are losses from another. Movement between the boxes is driven by physical transport (advection or vertical diffusion between cells or as point sources in specific grid cells). At any given model time-point, the constituents can be stored or can be transformed through biogeochemical processes (e.g., inorganic constituents become organic via photosynthesis). “Storage” in the box, i.e., the mass of the constituent in the box, is the value reported in the model solution for that grid cell. We utilized mass balance methods to investigate the changes in eutrophication state (O$_{2}$, pH) and rate (phytoplankton nitrogen uptake, NPP, respiration, and remineralization) variables resulting from terrestrial inputs and how these effects vary nearshore (depth <200 m) and offshore. These state and rate variables are also the starting point for investigations of effects of physical transport processes on coastal biogeochemistry, described in the subsequent section.

#### Nitrogen cycle

Human activities have augmented the coastal export of DIN, including NO$_{3}^{-}$ and NH$_{4}^{+}$), and this DIN augments primary productivity^7,58^. For this reason, we calculated two types of DIN mass balances, where $S_{DIN}$ represents sources and sinks from biogeochemical processes: 1) changes in subregional DIN loading and 2) net change in the phytoplankton DIN uptake rate, which is equivalent to photosynthesis also expressed as net primary production (NPP): $J^{DIN}= J^{NO_3^- }+ J^{NH_4^+}$

1 $$\begin{aligned} \partial {DIN}/\partial {t} = -\nabla \cdot (u\,DIN) + \partial _{z} (k_{v} \, \partial _{z} DIN) + S_{DIN} \end{aligned}$$

The advection term $-\nabla$ $\cdot$ ($u\,$DIN) is computed using the third-order upwind scheme^59^, and the diffusion term $\partial _{z} (k_{v} \, \partial _{z} DIN$ is parameterized by the KPP boundary-layer scheme^60^.

#### Oxygen cycle

The O$_{2}$ mass balance analysis includes physical transport and biogeochemical processes, where $S_{O_{2}}$ represents sources and sinks from biogeochemical processes including surface air-sea flux, photosynthesis, non-grazing mortality, grazing mortality, water-column remineralization, sediment remineralization, NH$_{4}^{+}$) oxidation, and nitrification (Eq. A9 in Deutsch et al.^19^):

2 $$\begin{aligned} \partial {O_{2}}/\partial {t} = -\nabla \cdot (u\,O_{2}) + \partial _{z} (k_{v} \, \partial _{z} O_{2}) + S_{O_{2}} \end{aligned}$$

#### DIC cycle

DIC mass balance includes physical transport and biogeochemical processes ($S_{DIC}$) of air-sea flux, photosynthesis, $CaCO_{3}$ production, non-grazing mortality, grazing mortality, and water-column and sediment remineralization (Eq. A11 in Deutsch et al.^19^).

3 $$\begin{aligned} \partial {DIC}/\partial {t} = -\nabla \cdot (u\,DIC) + \partial _{z} (k_{v} \partial _{z} DIC) + S_{DIC} \end{aligned}$$

We perform a change assessment from the monthly averages for the sum of the physical transport terms and for each of the biogeochemical process terms. Monthly averages of the biogeochemical rates and transport fluxes are performed between 0 and 200 m water depth of the epipelagic layer.

### Investigations of mechanisms controlling fate and transport of anthropogenic nitrogen

We investigated the relative importance of three possible mechanisms responsible for spatial patterns, in particular hot spots of O$_{2}$ and pH loss: (1) nitrogen is advected offshore, where it fuels new production, (2) nitrogen fuels new production inshore, which is then advected offshore, and (3) biomass produced or advected offshore is recycled and is regenerated to inorganic forms. An important control on these processes is the balance of DIN and organic matter following cross-shelf transport and entrapment in eddies, versus transport in the main northward current. Transport via the northward current causes export from the Bight domain, while entrapment within eddies has the potential to increase DIN residence time and accumulate organic matter within the Bight.

In order to understand the relative importance of these three processes, we conducted several analyses: We (1) quantified the mean cross-shelf fluxes of DIN and algal organic nitrogen (ON) and partitioned those fluxes into two main transport terms: (a) mean currents and (b) transient eddies, which are chaotic by nature^61,62^, but are implicated in intensifying gradients of vertical flux, productivity, respiration, and export^52,63^, and (2) calculated the relative importance of nitrification and NH$_{4}^{+}$ production from recycled organic matter. Additional methods to support the diagnostics on eddy characterization and fate and transport of coastal plumes can be found in the Supplemental material (section: Investigation on transient eddy characteristics and transport patterns).

#### Mean Current versus transient eddy decomposition

We separate the total fluxes of tracers into mean and transient eddy components following a typical Reynolds decomposition (Eqn. 4), e.g., horizontal zonal advection, where u is the velocity component, and N the concentration of a biogeochemical tracer (e.g., NH$_{4}^{+}$).

4 $$\begin{aligned} \overline{uN} = \overline{u}\overline{N} + \overline{u^{\prime }N^{\prime }}. \end{aligned}$$

The overbar denotes the monthly mean, and the prime the deviation from this mean, which removes long term variations. The Reynolds decomposition is applied to the physical fluxes of biogeochemical tracers along the cross-shore direction. Total fluxes, uN, are estimated online in the model and saved at daily resolution. The mean component of the fluxes, uN, is calculated from daily mean velocities and tracer concentrations. Eddy fluxes, u’N’ are calculated by difference, based on Eqn. 4. We note that “eddy” is generally a term used for any kind of mesoscale and submesoscale activity.

#### Horizontal export of DIN and ON from the shelf

We calculate the transport across a vertical section that follows the 200 m isobath, a representative of the limit of the shelf domain^64^, to quantify the flux from nearshore to offshore. The calculation is done at every daily averaged velocity and nutrient concentration. For visualization purposes, the alongshore profiles are smoothed using a Gaussian filter with a 20 km horizontal window. The across-shore export from the shelf to offshore is presented as positive values, and the import to the shelf is negative (e.g., upwelling). We applied this calculation to total ON, NO$_{3}^{-}$ plus NO$_{2}^{-}$, and NH$_{4}^{+}$ fluxes separately. The total ON accounts for phytoplankton and zooplankton biomass plus DON.

### Model performance and uncertainty assessment

SCB ROMS-BEC model validation previously documented in detail the model performance relative to observations for physics, biogeochemistry and lower trophic ecosystem properties for the period of 1997–2001^8^. It demonstrated good to excellent performance in capturing key gradients (seasonal cycle, vertical distribution, spatial variability) of coastal and offshore physical variables, nutrients, O$_{2}$, pH, omega aragonite and Chl-a. We updated this skill assessment for the more recent period (August 2012–November 2017) as a check up on performance, then compared how the predicted magnitude of O$_{2}$ loss compared with two quantifiable sources of model uncertainty: (1) differences between observations and model predictions and (2) intrinsic variability that arises from stochastic ocean processes, in order to answer the question *“does the modeled signal of anthropogenic change emerge from the noise of quantifiable sources of model uncertainty?”* We focus on O$_{2}$ for this quantifiable uncertainty assessment, because pH observations based on potentiometric methods have poor precision^8^.

For the model skill assessment, we use the same metrics and performance criteria^65^ detailed in the previous version of the model evaluation. For pH, in this updated assessment, we use newly available in situ bottle data that has a detection limit of $+/-$0.05. These in situ bottle data are available for 8 sampling events in 2015 and 2016. This is in contrast to data used in^8^, which were from potentiometric sensors that have poor precision ($+/-$0.4). Performance is summarized in Taylor diagrams^66^ using the R package developed by Carslaw et al.^67^ on which we color-coded a matching criterion^66^ as cited in Allen et al.^65^.

The difference between observational data and model predictions is a quantitative measure of uncertainty, which arises from multiple sources, including inaccurate model formulation, parameterization, numerical approximations, boundary forcing, observational error, spatial/temporal mismatch, etc. Differences in ANTH-CTRL are scale-dependent, so we endeavoured to put the magnitude of this change on the same scale at which data-model differences are assessed (on the vertical scale, in monthly averaged model virtual profile data). From the offshore subregion where the model predicted recurring, substantial O$_{2}$ losses, we selected five CalCOFI monitoring stations, then assessed the absolute magnitude of data-model differences relative to the magnitude of predicted O$_{2}$ loss (ANTH-CTRL) at that station, selecting one period of maximum O$_{2}$ loss using the summer 2013 sampling event for graphical illustration.

To assess intrinsic variability, two additional ANTH scenarios were generated for the period of September 2012-November 2013. The scenarios were set up using forcing that are identical to ANTH, but with perturbed initial conditions (ANTH2 and ANTH3) for which we added a random uniform variability to all state variables, including physical and biogeochemical variables in the initial condition. The perturbation is created by averaging random 3 days among the period that span between August 15 and September 15, 2012 from the original ANTH scenario.

We assess O$_{2}$ change in ANTH - CTRL, then compare the magnitude of that change with that from each pair of ANTH simulations (ANTH-ANTH2, ANTH-ANTH3, ANTH2-ANTH3) in order to determine whether a signal of anthropogenic change emerges from the noise of intrinsic variability.

A depth of 50 m was nominally chosen to do a quantitative comparative analysis of the magnitude of ANTH-CTRL versus intrinsic variability and data-model uncertainty for the average of the 5 CalCOFI stations for the summer 2013.

The assessment of uncertainty focuses mainly on intrinsic oceanic sub annual variability that emerges within the model domain. We note that other sources could occur outside the SCB domain, for which a new set of nested simulations would be required to quantify. We leave this more comprehensive quantification of intrinsic variability to future studies.

## Supplementary Information

Supplementary Information.

## Data Availability

The datasets used and/or analysed during the current study are available from the corresponding author on reasonable request.

## Code avilability

Code is available in Kessouri et al.^68^. Local land-based and atmospheric data can be found in Sutula et al.^20^. Due to the size of the output, model outputs are available from the authors upon request.
