# Author Correction: Cross-shore transport and eddies promote large scale response to urban eutrophication

**DOI:** 10.1038/s41598-024-74723-8

**Published:** 2024-10-02

**Authors:** Fayçal Kessouri, Martha A. Sutula, Daniele Bianchi, Minna Ho, Pierre Damien, James C. McWilliams, Christina A. Frieder, Lionel Renault, Hartmut Frenzel, Karen McLaughlin, Curtis Deutsch

**Affiliations:** 1 Department of Biogeochemistry, Southern California Coastal Water Research Project, 3535 Harbor Blvd, Suite 110, Costa Mesa, CA 92626 USA; 2 Department of Atmospheric and Oceanic Sciences, University of California Los Angeles, Los Angeles, CA 90095 USA; 3 Laboratoire d’Études en Géophysique et Océanographie Spatiale, IRD, CNRS, CNES, UPS, Toulouse, 31400 France; 4 School of Oceanography, Seattle, WA 98195 USA; 5 Department of Geosciences, High Meadows Environmental Institute, Princeton University, Princeton, NJ 08544 USA; 6 Present Address: CICOES, University of Washington and NOAA PMEL, Seattle, WA 98105 USA

Correction to: *Scientific Reports*10.1038/s41598-024-57626-6, published online 27 March 2024

The original version of this Article contained a repeated error in the area size stated in square kilometres.

As a result, in the Abstract, 

“This phenomenon persists for 4 to 6 months of the year over an area of 278,40 km^2^ (∼30% of SCB area).”

now reads:

“This phenomenon persists for 4 to 6 months of the year over an area of 27,840 km^2^ (∼30% of SCB area).”

In addition, in the Results, under the subheading ‘Effects of land-based nutrient inputs on phytoplankton biomass, oxygen, and pH’,

“The area impacted by these biogeochemical changes is approximately 278,40 km^2^ in size.”

now reads:

“The area impacted by these biogeochemical changes is approximately 27,840 km^2^ in size.”

The original Article has been corrected.

