# Supplementary material for: Cross-shore transport and eddies promote large scale response to urban eutrophication
Source: Sci Rep. 2024 Mar 27;14:7240. doi: 10.1038/s41598-024-57626-6 (PMC11350003; doi:10.1038/s41598-024-57626-6)
Supplement: Supplementary file 1 — Supplementary Information. [file 41598_2024_57626_MOESM1_ESM.pdf]

Supplemental information

Investigation on transient eddy characteristics and transport patters

We investigate the type of circulation responsible for material transport by using the Okubo-Weiss parameter, a measure of the relative importance of formation and rotation, and the relative vorticity, a measure of the angular momentum of water parcels (Fig. S2). Both quantities were averaged over the 0-80 m layer and used to characterize the form of submesoscale activity associated with the horizontal flux of NH_4^+ across the shelf. Positive Okubo-Weiss index defines coherent vortices, while negative defines straining regimes. Vorticity helps identifying cyclones from anti-cyclones. A probability distribution function (PDF) of the horizontal flux is presented by type of regime: cyclones, anti-cyclones, and straining/steering. The latter represents the dynamics encapsulating transport in jets, filaments, and fronts around and between vortices. The typical size of those features that the model could resolve is generally between 1-10 km. Cross-shore transport fluxes were analyzed by applying a mean versus eddy decomposition of the inorganic and organic nitrogen horizontal transport. We also conducted a plume tracking analysis using Lagrangian drifters to track the extent of wastewater plumes from the point sources to the offshore domain.

Lagrangian trajectories. Use of Lagrangian drifters[69,70] was intended to illustrate the dominant transport mechanisms that influence the fate of anthropogenic nitrogen over time scales of days to a month, which spans the turnover time of phytoplankton DIN update, remineralization, and turnover. As defined in[69], the drifters algorithm resolves the equation

$d\vec{X}(t)) = \vec{U}(t, \vec{X}$

where $\vec{X}(x,y,z)$ is the particle position and $\vec{U}(u,v,w)$ serves as the Eulerian velocity. We applied drifters to track the transit of a conservative tracer applied to each of the 19 ocean outfalls and 75 rivers during a 2-month period (October 1st, 2013 to November 30, 2013) using hourly outputs of ROMS velocities, the fall period in which subsurface O_2 and pH most frequently occurs. Five neutrally buoyant particles were seeded every 6 hours at each grid point of coastal input (200 locations total = 4000 particles per day) during the entire experiment (2 months   1464 hours). The particles were seeded at the depth of their original input, i.e., rivers at the surface and plumes at their designated outfall depth. We utilized a two-month averaged heat map of the visitation′s frequency and maps of snapshots of the trajectories to illustrate the transport of the plumes in the coast and offshore.

The Lagrangian tracer studies demonstrate how conservative tracers of riverine and outfall plumes connect with mean northward current and persistent eddies, see Fig S4 of particular note, 1) South of the Channel Islands, the northwestward branch of the coastal current transports the particles out from the shelf to direct them away from the coast and accumulate them in the re-circulation that will then join the southern branch of the CCS; 2) plume materials show clear cross-shelf transport and B), 3) plumes can travel great distances, e.g., the southern plumes originating near San Diego and the northern plumes from Palos Verdes join in a shear that forms between next to Santa Catalina Island and 4) a clear and consistent accumulation of particles suggests that they are trapped in the persistent eddy southeast of Santa Catalina Island.

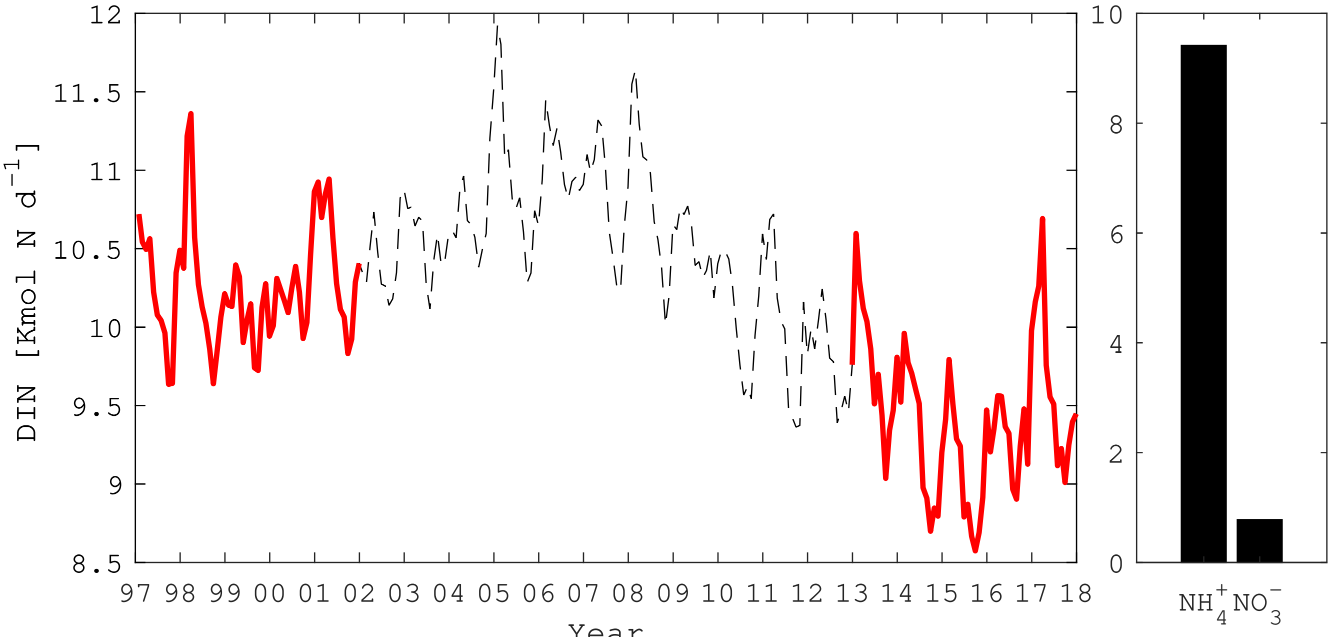

Figure S1. Time-series of total nitrogen export from the coast to the ocean between 1997 and 2017. The red line highlights the period of the previous (1997-2000) and 2013-2017 simulations. The time-series shows a reduction in the total load of nitrogen since 2008.

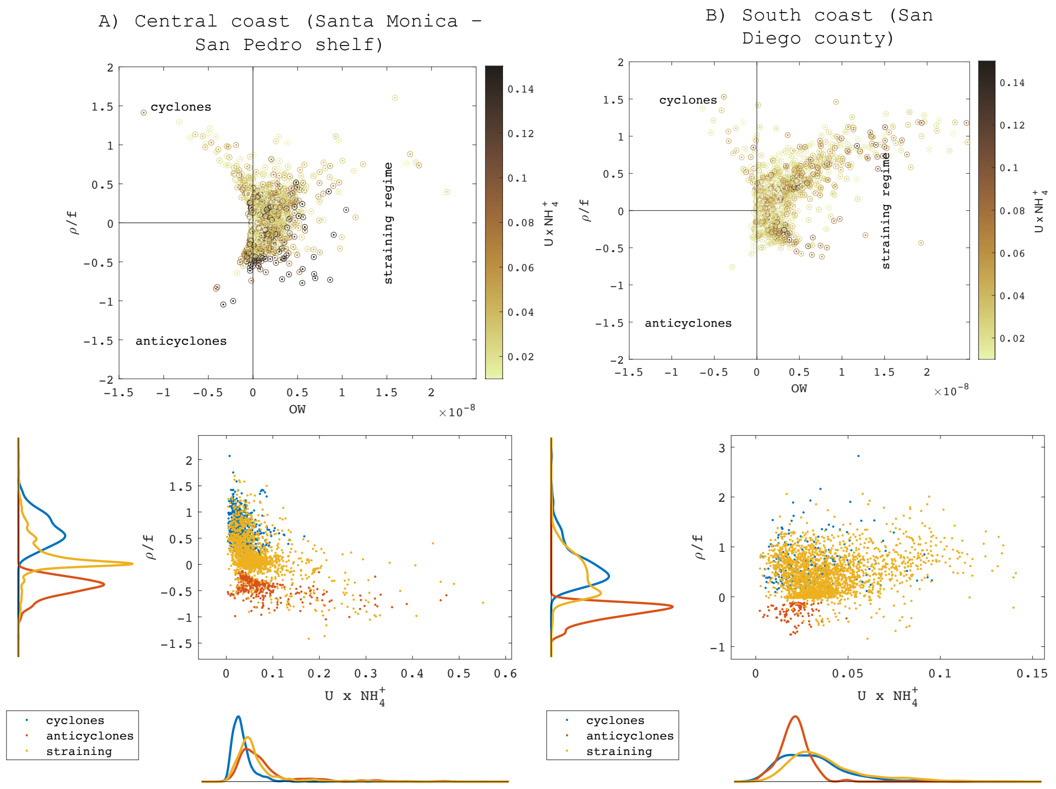

Figure S2. upper panels: Total cross shore flux of ammonium in the upper 0-80 m layer plotted in an Okubo-Weiss relative vorticity diagram (see SI). This diagram defines the type of physical regime encountered by the parcel of water. For visualization purposes, we subsampled randomly 5000 samples from the 5 years daily outputs. A) for central coast along Santa Monica Bay and San Pedro shelf, latitudes = [33.7 to 34.05°N]. B) for San Diego shelf latitudes = [32.2 to 33.2°N]. Lower panels: scatter plot with marginal histogram for the cross-shore flux and relative vorticity provided by type of regime: cyclones, anticyclones, and straining form. Arrows point out the regimes with the highest flux of ammonium. Generally, high fluxes can be simulated in all three regimes. There are some dominant regimes in each part of the domain, in A) for straining regime associated with anticyclones, in B) for straining regime associated with cyclones.

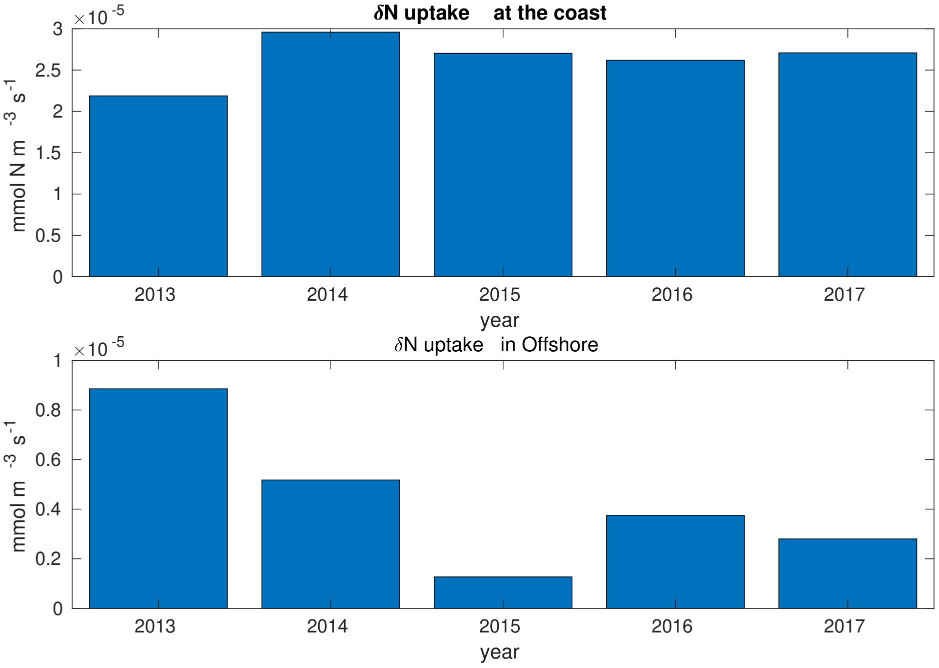

Figure S3. Change assessment in the nitrogen uptake in the coastal band (upper panel) and offshore (lower panel) averaged by year. It shows the consistency in the amplification in the biogeochemical terms of the mass balance.

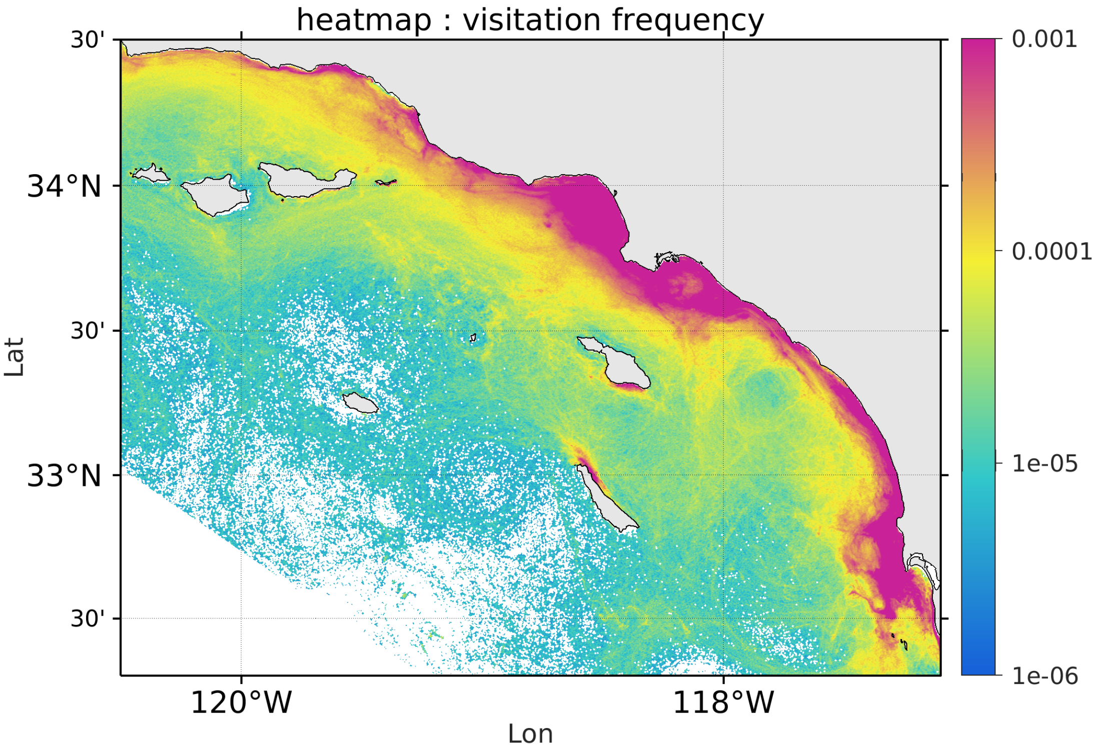

Figure S4. The heat map represents visitation frequency of the particles released from ocean outfalls and rivers for 2 months between October and November 2013. The maps shows highest visitation in the coastal area. Offshore the highest visitations took place in the branch toward south Santa Catalina Island, and toward south Channel Islands. This map has been generated using the version 2022 of the programming software Matlab[56] and and the mapping toolbox created by Piretzidis et al., 2016[57].
